# Frozen melanoma tissues yield extracellular vesicles with preserved diagnostic and immunogenic properties

**DOI:** 10.1186/s12916-026-04923-8

**Published:** 2026-05-21

**Authors:** Daniele D´Arrigo, Kyong-Su Park, Cecilia Lässer, Georgios Panagiotis Sigalas, Emma Symonds, Ornella Urzí, Camilla Locatelli, Roger Olofsson Bagge, Jan Lötvall, Rossella Crescitelli

**Affiliations:** 1 Krefting Research Centre, Department of Internal Medicine and Clinical Nutrition, Institute of Medicine, Sahlgrenska Academy, University of Gothenburg, Gothenburg, Sweden; 2 Regenerative Medicine Technologies Laboratory, Ente Ospedaliero Cantonale, Bellinzona, Switzerland; 3 IRCCS Humanitas Research Hospital, Rozzano, Milan, Italy; 4 Sahlgrenska Center for Cancer Research, Department of Surgery, Institute of Clinical Sciences, Sahlgrenska Academy, University of Gothenburg, Gothenburg, Sweden; 5 St. Anna Children’s Cancer Research Institute (CCRI), Vienna, Austria; 6 Department of Surgery, Sahlgrenska University Hospital, Region Västra Götaland, Gothenburg, Sweden

**Keywords:** fresh tissue, frozen tissue, melanoma, exosomes, tissue-derived extracellular vesicles, extracellular vesicle biomarkers, cancer immunotherapy

## Abstract

**Background:**

Extracellular vesicles (EVs) isolated from tumor tissues carry disease-associated proteins and surface antigens, making them promising candidates for cancer diagnostics and immunotherapy. For EV-based approaches to reach clinical application, it is essential that functional EVs can be obtained from clinically accessible materials, including cryopreserved tumor tissues stored in biobanks. Whether cryopreservation alters EV integrity and function remains unclear. This study evaluates whether EVs derived from cryopreserved tumor tissues retain key molecular and biological properties required for diagnostic and therapeutic use.

**Methods:**

EVs were isolated from human metastatic melanoma tissues processed immediately after surgical resection (fresh) or after storage at − 80 °C (frozen), using a protocol consistent with standard biobank procedures. EV isolation was performed through ultracentrifugation followed by an iodixanol density cushion. The resulting EVs were characterized by transmission electron microscopy, nanoparticle tracking analysis, and mass spectrometry to assess EV morphology, purity, and molecular composition. The diagnostic potential was evaluated by examining the presence of previously identified cancer-associated membrane proteins. Furthermore, therapeutic potential was assessed in vivo by co-administering EVs from fresh or frozen melanoma tissues with synthetic bacterial vesicles and evaluating their effects on tumor growth in melanoma-bearing mice.

**Results:**

EVs from fresh and frozen tissues showed similar morphology, size distribution, yield, and purity. Moreover, the protein composition, including cancer-associated markers such as MT-CO2, COX6c, SLC24A22, HLA-DR, and Erlin2, was highly consistent between EVs derived from fresh and frozen tissues, with no relevant enrichment of intracellular or mitochondrial contaminants in frozen-derived EVs. Functionally, EVs from cryopreserved tissues combined with synthetic bacterial vesicles significantly inhibited tumor progression in vivo, demonstrating antitumor effects comparable to those of EVs from fresh tissues.

**Conclusions:**

Our results validate cryopreserved tissues as a reliable source of functional EVs, comparable to fresh tissues. This supports the potential use of existing biobanks for retrospective EV-based biomarker discovery and functional research.

**Graphical abstract:**

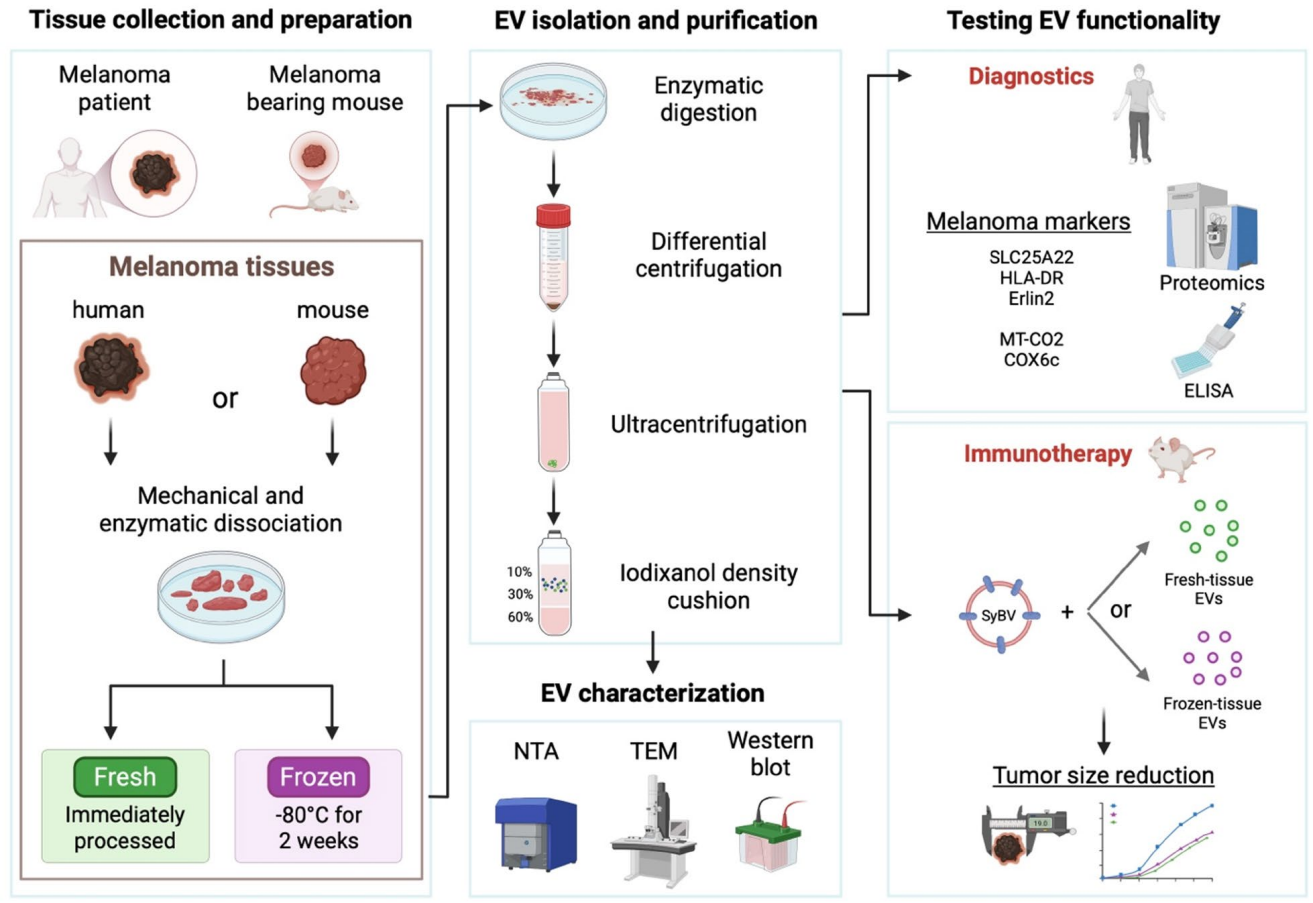

**Supplementary Information:**

The online version contains supplementary material available at 10.1186/s12916-026-04923-8.

## Background

Tissue homeostasis relies on complex cell-cell interactions, in which extracellular vesicles (EVs) play a central role by mediating intercellular signaling [1–4]. EVs are implicated in tumor progression and are emerging as promising disease biomarkers, as they reflect the molecular state of their cells of origin [5, 6]. In addition, EVs could exhibit immunogenic properties, supporting their potential as anticancer and immunotherapeutic agents [7, 8]. Owing to these features, EVs are increasingly recognized as versatile tools for cancer diagnosis and therapy.

In oncology, EVs isolated from biofluids and solid tissues are being explored as biomarkers for early detection, disease monitoring, and prognosis, as they carry tumor-specific proteins, lipids, and nucleic acids [9, 10]. For example, exosomal PD-L1 has been proposed as a predictor of response to immune checkpoint blockade, while EV-associated proteins such as GPC-1 and tumor-enriched miRNAs show diagnostic potential [9, 10]. Moreover, EVs within tumor tissues can carry tumor antigens [11–13], highlighting their potential for cancer immunotherapy.

EV-based diagnostics most commonly rely on EVs isolated from biofluids such as blood, urine, and saliva [14, 15]. However, these EVs often show limited diagnostic robustness due to dilution by non-disease EVs [15–18]. In contrast, EVs derived from solid tumors are highly concentrated, disease-specific, and reflect the tumor microenvironment, making them a valuable source for biomarker discovery [19, 20]. We previously showed that melanoma tissue-derived EVs are enriched in mitochondrial proteins such as MT-CO2, COX6c, SLC24A22, HLA-DR, and Erlin2, and two of these (MT-CO2, COX6c) were also elevated in the plasma of patients, supporting their translational relevance [19]. Furthermore, we recently showed that tumor tissue-derived EVs combined with synthetic bacterial vesicles (outer membrane vesicles modified to eliminate membrane and cytosolic components) elicit immune-dependent tumor growth inhibition in melanoma models [21, 22], indicating their potential as neoantigen sources for cancer vaccination [21].

Despite this promise, translational studies using tissue-derived EVs remain limited due to methodological challenges and a lack of standardization, as protocols for EV isolation from fresh solid tissues have emerged only recently [19, 23–26]. Even fewer studies have explored frozen tissues as a source of EVs, despite the widespread availability of biobanked specimens [25, 27, 28]. Recent studies have begun to explore the feasibility of isolating EVs from frozen tissues, reporting that some key EV features are preserved under specific conditions [29].

This raises the important question of whether cryopreservation affects the quality, purity, and functionality of tissue-derived EVs.

Although cryopreserved tissues are more prone to cellular disruption [30], EVs themselves are structurally resilient and may remain intact after freezing. Their membrane curvature, lipid composition, and associated proteins likely contribute to their stability at low temperatures. EVs < 150-200 nm exhibit high membrane curvature, which may increase their resistance to deformation [31, 32]. Membrane lipids, including cholesterol, sphingolipids, and glycerophospholipids, help maintain stability during freezing [33, 34], while proteins such as lactadherin may further support membrane integrity [35]. Notably, EVs preserved within their native tissue environment appear better maintained than isolated EVs stored in buffers [29, 36–39]. Taken together, these findings support the use of frozen tissues as a valid source of EVs. However, most studies relying on frozen tissues lack rigorous comparisons with fresh counterparts, raising concerns about potential artifacts introduced during the freezing process and the reliability of functional assessments [20, 25–28, 40–46].

To address these issues, we hypothesized that EVs isolated from fresh and frozen human melanoma tissues are comparable in terms of protein cargo and immunotherapeutic properties. The freezing protocol involved placing tissue samples in a precooled rack and storing them at − 80 °C for two weeks. This approach is simple and widely used for long-term biobanking [47–49]. We isolated EVs from matched fresh and frozen tissues and evaluated their purity, proteomic profiles, diagnostic biomarker content, and in vivo immuno-oncology potential. These analyses were conducted to determine whether cryopreserved tissues can serve as a reliable source of EVs for cancer diagnostics and immunotherapy development.

## Methods

### Patient information

Melanoma tissues were collected from nine patients diagnosed with stage III or IV melanoma during surgical resection at the Department of Surgery at Sahlgrenska University Hospital between December 2021 and November 2025. The biological material obtained for this study included two cutaneous metastases, six lymph node metastases, and one intestinal metastasis, with weights ranging from 0.2 g to 4.5 g. Patient demographics are listed in Table 1.

**Table 1:** Patient demographic information

Patient	Type of metastasis	Sex	Age	TNM-stage	Sample used for
1	Cutaneous metastasis (in-transit)	Male	59	Stage III	Cell analysis: single cell isolation, cytospin, EV analysis: TEM, NTA, LC-MS/MS
2	Lymph node metastasis	Male	48	Stage IV	Cell analysis: single cell isolation, cytospin, EV analysis: NTA
3	Lymph node metastasis	Male	56	Stage IV	Cell analysis: single cell isolation, cytospin, EV analysis: WB, ELISA, NTA, LC-MS/MS
4	Skin metastasis	Male	56	Stage IV	Cell analysis: single cell isolation, cytospin, EV analysis: TEM, NTA, LC-MS/MS
5	Lymph node metastasis	Male	81	Stage III	Cell analysis: single cell isolation, cytospin, EV analysis: WB, ELISA, NTA, LC-MS/MS
6	Lymph node metastasis	Female	63	Stage III	EV analysis: WB, ELISA, NTA
7	Lymph node metastasis	Male	74	Stage III	EV analysis: WB, ELISA, NTA
8	Lymph node metastasis	Male	77	Stage III	EV analysis: WB, ELISA, NTA
9	Intestine metastasis	Male	83	Stage IV	EV analysis: WB, ELISA, NTA

TEM= Transmission Electron Microscopy

NTA= Nanoparticle Tracking Analysis

LC-MS/MS= Liquid Chromatography Tandem Mass Spectrometry

WB= Western Blot

### Cell cultures and PBMC

The human skin melanoma cells MML-1 (CSL, Eppelheim, Germany) were cultured in RPMI-1640 (Cytiva, Loga, UT, USA) supplemented with 10% FBS (Sigma-Aldrich, St. Louis, MO), 100 units/mL penicillin, 100 mg/mL streptomycin (Cytiva), and 2 mM L-glutamine (Cytiva). The human hepatocarcinoma cell line HepG2 was cultured in DMEM (Thermo Fisher Scientific, Waltham, MA) supplemented with 10% FBS (Sigma-Aldrich) and 1% penicillin/streptomycin (Cytiva). Cells were maintained at 37 °C with 5% CO_2_ for 2 weeks before being lifted from flasks using TrypLE Express Enzyme (Thermo Fisher Scientific) and stained for FACS analysis.

Peripheral blood mononuclear cells (PBMCs) were isolated from buffy coats collected in K2E EDTA tubes from healthy donors using Ficoll-Paque Plus (Cytiva) density gradient centrifugation.

### Animals

Wild-type C57BL/6 mice (6 weeks old) were obtained from Charles River. The mice were housed at the Experimental Biomedicine (EBM) facility at the University of Gothenburg, Sweden. The study received approval from the local Animal Ethics Committee in Gothenburg, Sweden (Dnr 5.8.18–10595/2023) and was conducted in accordance with institutional animal use and care guidelines.

### Tissue handling and freezing

Immediately after tumor resection, both human (*N* = 9) and mouse melanoma (*N* = 3) tissues were weighed and divided into two equal parts. Each part was placed in RPMI-1640 (Sigma-Aldrich) and cut into approximately 2 mm × 2 mm × 2 mm pieces with a scalpel. One part was processed immediately as fresh samples, while the other was transferred to 1.5 mL conical cryovials on dry ice to minimize air bubble formation (frozen samples). To snap-freeze the samples, which is less damaging to EV integrity, the cryovials were placed in a metallic rack (Corning, Corning, NY) that had been pre-cooled in an ice pan filled with dry ice. They were incubated until fully frozen to prevent slow freezing, which could harm EVs [39]. These tissues were stored at − 80 °C for 2 weeks before further processing. The procedure is shown in Fig. 1.1

### EV isolation from human and mouse melanoma metastatic tissue

**Fig. 1:**
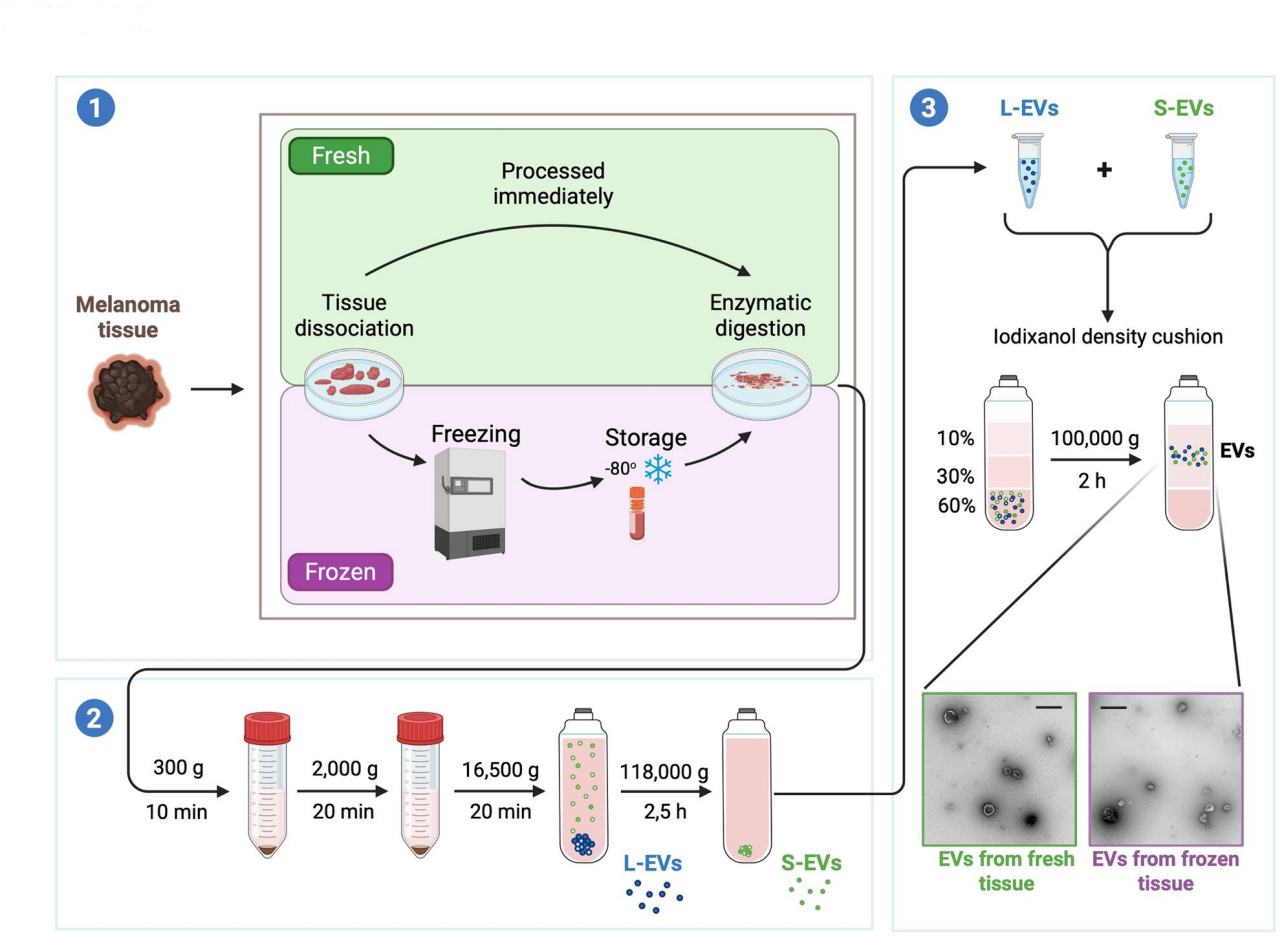
Graphical representation of the experimental design. The melanoma tissues were divided into two parts after collection. One part was immediately processed to isolate fresh EVs. The second part was dissected into small tissue pieces, frozen in dry ice, and stored at − 80 °C for two weeks before being processed to isolate EVs from cryopreserved tissues **1**). Isolation was performed through a combination of differential centrifugations **2**) and density cushion **3**). L-EVs: large EVs; S-EVs: small EVs. Scale bar of TEM pictures is 500 nm

EVs from both human (*N* = 9) and mouse melanoma tissues (*N* = 3) were isolated immediately from fresh samples, whereas frozen tissues were stored for two weeks and subsequently thawed on ice prior to processing to preserve their structural and functional integrity as much as possible [50, 51]. Thawing on ice is also recommended in MISEV guidelines to better preserve the structural and functional integrity of the EVs [50, 51]. The same protocol described by Crescitelli et al. [24] was used to isolate EVs from both fresh and frozen tissues, with minor modifications. In brief, just after the resection or thawing, the tissue was dissociated into small pieces (~ 2 mm × 2 mm × 2 mm) and then digested for 30 min at 37 °C with Collagenase (2 mg/mL, Roche, Basel, Switzerland) and DNase I (400 U/mL, Roche). The digested tissue fragments were filtered through a 70 μm filter. The flow-through was used for EV isolation, while the remaining parts in the filter (tissue pieces collected after enzymatic treatment and filtration) were further processed as described in paragraph 2.7. The procedure is schematized in Fig. 1. Potential contaminants from cells and debris were removed by two centrifugation steps (300 × *g* for 10 min and 2,000 × *g* for 20 min). The supernatant was then transferred to a polypropylene Quick Seal centrifuge tube (Beckman Coulter, Brea, US) and centrifuged at 16,500 × *g* (14,500 rpm) at 4 °C for 20 min using a Type Ti 45 fixed-angle rotor (Beckman Coulter, k-factor 1.275) to isolate large EVs. The pellet was then resuspended in PBS and stored at − 80 °C until further processing, while the supernatant was further centrifuged at 118,000 × *g* (38,800 rpm; Type Ti 45 fixed-angle rotor, Beckman Coulter, k-factor 178) for 2.5 h at 4 °C to isolate small EVs, which were resuspended in PBS and stored at − 80 °C.

### Iodixanol density cushion

Both human and mouse tissue-derived EVs isolated by ultracentrifugation were further purified using iodixanol density cushion centrifugation as described by Crescitelli et al. [24], with minor modifications. Both large and small EVs were thawed on ice and combined. The EVs were then mixed with 3 mL of 60% Optiprep (Sigma Aldrich) at the bottom of an Ultra-Clear centrifuge tube (Beckman Coulter), and 4 mL of 30% Optiprep and 4 mL 10% Optiprep were layered on top. Samples were ultracentrifuged at 100,000 × *g* (28,000 rpm) in a SW 41 Ti Swinging-Bucket Rotor (Beckman Coulter, k-factor 145) for 2 h at 4 °C. The samples were collected from the interface of the 10% and 30% iodixanol layers and stored at − 80 °C.

### Tissue dissociation and single-cell isolation

The remaining human tissue pieces after enzymatic treatment and filtration of the fresh sample were processed using a human tumor dissociation kit (Miltenyi Biotec, Bergisch Gladbach, Germany) according to the manufacturer’s instructions to produce a single-cell suspension. Briefly, the samples were transferred to a plastic tube containing a knife and placed in the gentleMACS Dissociator (Miltenyi Biotec) for further dissociation, then incubated with enzymes for 30 min at 37 °C under continuous rotation. These steps were repeated three times, after which the dissociated samples were filtered through a 70 μm filter. Following centrifugation at 300 × *g* for 7 min, the cell pellet was resuspended in RPMI-1640 (Sigma-Aldrich). The cell concentration was determined using the trypan blue exclusion method.

### Flow cytometry analysis of cells isolated from fresh metastatic melanoma tissues

The cellular composition of human solid tumor tissues was analyzed using flow cytometry. The expression of these markers was initially assessed in control samples, including MML-1 cells, HepG2 cells, and PBMCs. Single cells were incubated with human IgG (Sigma-Aldrich) at 4 °C for 15 min to block nonspecific binding sites. Subsequently, the cells were stained with a viability stain kit (LIVE/DEAD Fixable Aqua Dead Cell Stain Kit, Invitrogen, Life Technologies Corp, Eugene, OR) and antibodies targeting cell-specific surface antigens, specifically anti-CD3-APC (final concentration 20 µg/mL, clone SK7) and anti-CD45-PerCP (final concentration 2.5 µg/mL, clone 2D1), both from BD Biosciences (San Jose, CA). After 30 min of incubation at 4 °C in the dark, the cells were washed with wash buffer (1% FBS in PBS), then fixed and permeabilized with the eBioscience Foxp3/Transcription Factor Staining Buffer Set (Invitrogen) for 1 h at 4 °C in the dark. Antibodies against SOX-10-PE (final concentration 2 µg/mL, clone SP267) and isotypic control-PE (final concentration 2 µg/mL, clone EPR25A), both from Abcam, Cambridge, UK) were added and incubated for another hour at 4 °C in the dark. Following washing, the cells were analyzed with a BD FACSVerse Flow Cytometer equipped with BD FACSSuite software (BD Biosciences), and the data were analyzed with FlowJo software (Tree Star Inc., Ashland, OR). The antibody signals were gated based on control samples prepared using the FMO method, in which each control lacked a specific marker (Additional file 1: Fig. S1). Only live cells were included in the gating. Additionally, 50,000 cells were placed in a single layer on a glass slide using a cytocentrifuge and stained with Hemacolor Rapid (Merck Millipore, Darmstadt, Germany) according to the manufacturer’s instructions.

### Particle measurement

The number of particles isolated from both human and mouse melanoma tissues was measured using a ZetaView PMX110 instrument (Particle Metrix, Meerbusch, Germany). Measurements were taken at 11 positions, with video quality set to medium and camera sensitivity set to 80. The software automatically assessed and included the sample chamber temperature in the calculation. Data were analyzed using ZetaView analysis software (version 8.2.30.1) with a minimum size of 5, a maximum size of 5,000 and a minimum brightness of 20.

### Protein measurement

Human tissue-derived EVs resuspended in PBS were used to measure EV protein concentration using a Qubit device (Thermo Fisher Scientific) according to the manufacturer’s instructions. The protein concentration of EVs isolated from mouse tissues was measured using the Pierce BCA Protein Assay Kit (Thermo Fisher Scientific) following the manufacturer’s instructions. Absorbances were read at 562 nm on a Varioskan LUX Multimode Microplate Reader (Thermo Fisher Scientific).

### Western blot analysis

For the western blot analysis of human melanoma-derived EVs, proteins were extracted with RIPA buffer (Thermo Fisher Scientific), and 10 µg were then loaded and separated on precast 4-20% polyacrylamide Mini-PROTEAN TGX gels (Bio-Rad Laboratories, Hercules, CA). The proteins were then transferred to PVDF membranes (Bio-Rad Laboratories), which were incubated in EveryBlot Blocking Buffer (Bio-Rad Laboratories) for 5 min at RT to block nonspecific binding. The following primary antibodies diluted in EveryBlot Blocking Buffer (Bio-Rad Laboratories) were then added to the membrane and incubated overnight at 4 °C: anti-Calnexin (final concentration 0.03 µg/mL, clone C5C9, Cell Signaling Technology, Danvers, MA), anti-Mitofilin (final concentration 2 µg/mL, clone AB-2547893, Invitrogen), anti-ADAM10 (final concentration 1 µg/mL, clone 163003, R&D Systems, McKinley Place, MN, in non-reducing conditions), anti-CD63 (final concentration 0.05 µg/mL, clone H5C6, BD Biosciences, in non-reducing conditions), anti-Flotillin-1 (final concentration 0.58 µg/mL, clone EPR6041, Abcam), anti-CD9 (final concentration 0.1 µg/mL, clone MM2/57, Merck-Millipore, in non-reducing conditions), anti-CD81 (final concentration 1 µg/mL, clone M38, Abcam, in non-reducing conditions), anti-MT-CO2 (final concentration 1 µg/mL, clone 12C4F12, Thermo Fisher Scientific), and anti-COX6c (final concentration 2 µg/mL, clone 3G5F7G3, Abcam). After three washes, the secondary antibodies, diluted in EveryBlot Blocking Buffer (Bio-Rad Laboratories), were added to the membrane and incubated for 1 h at room temperature. The secondary antibodies used were anti-rabbit IgG (horseradish peroxidase (HRP) conjugated, final concentration 0.1 µg/mL, Harlan Sera-Lab, Loughborough, UK) and anti-mouse IgG (HRP conjugated, final concentration 0.1 µg/mL, Harlan Sera-Lab). Finally, the membranes were imaged and analyzed using the SuperSignal West Femto maximum Sensitivity Substrate (Thermo Fisher Scientific) and a ChemiDoc Imaging System (Bio-Rad Laboratories).

### ELISA

For the direct ELISA assays, each human melanoma-derived EV formulation (0.2 µg per well) was coated on a black 96-well plate overnight at 4 °C. EVs isolated from HEK293 cell supernatant were used as a negative control, as they have previously been shown to be negative for MT-CO2 and COX6c [19]. The plate was blocked with 1% BSA in PBS for 1 h, then incubated with anti-MT-CO2 (final concentration 5 µg/mL, clone 12C4F12, Thermo Fisher Scientific) or anti-COX6c (final concentration 5 µg/mL, clone 3G5F7G3, Abcam) diluted in 1% BSA in PBS for 2 h. After washing, the HRP-conjugated anti-mouse antibody (1:5,000 dilution, Harlan Sera-Lab) was diluted in 1% BSA in PBS and incubated for 1 h at room temperature. Luminescent signal was detected using the BM Chemiluminescence ELISA Substrate (BD Biosciences, San Jose, CA).

### Transmission electron microscopy (TEM)

The morphology of EVs isolated from both human and mouse melanoma tissues was examined by negative staining as previously described [23, 24]. Briefly, 5 µg of the sample was placed onto a glow-discharged 200-mesh formvar/carbon copper grid (Electron Microscopy Sciences, Hatfield Township, PA) for 5 min. After two washes with water, the EV samples were fixed in 2.5% glutaraldehyde and washed again before staining. To stain the specimens, 2% uranyl acetate was incubated for 1.5 min, and the negative-stained EVs were examined on a Talos L120C electron microscope (Thermo Fisher Scientific) at 120 kV with a CCD camera.

### Sample preparation and digestion for proteomic analysis

The samples and the reference pool, a representative sample containing aliquots from all the samples, were processed using a modified filter-aided sample preparation (FASP) method [52]. In brief, human melanoma-derived EVs (40 µg) were reduced with 100 mM dithiothreitol at 60 °C for 30 min, transferred to Microcon-30 kDa centrifugal filter units (Merck), and washed multiple times with 8 M urea and once with digestion buffer (50 mM TEAB, 0.5% sodium deoxycholate). They were then alkylated with 10 mM methyl methanethiosulfonate in digestion buffer for 30 min at room temperature. The samples were digested with trypsin (Pierce MS-grade trypsin, Thermo Fisher Scientific, 1:100 ratio) at 37 °C overnight, with an additional portion of trypsin added and incubated for another 2 h. Peptides were collected via centrifugation and labeled using tandem mass tag (TMT) 11-plex isobaric mass tagging reagents (Thermo Fisher Scientific), as shown in Additional file 10: Table S1, following the manufacturer’s instructions. The samples were combined into one TMT set, and sodium deoxycholate was removed by acidification with 10% TFA. The TMT set was then purified using High Protein and Peptide Recovery Detergent Removal spin columns and Pierce peptide desalting spin columns (both from Thermo Fisher Scientific) according to the manufacturer’s instructions before proceeding to basic reversed-phase chromatography (bRP-LC) fractionation. Peptide separation was performed using a Dionex Ultimate 3000 UPLC system (Thermo Fisher Scientific) with a reversed-phase XBridge BEH C18 column (3.5 μm, 3.0 × 150 mm, Waters Corporation, Milford, Massachusetts, MA), employing a gradient from 3% to 100% acetonitrile in 10 mM ammonium formate at pH 10.00 over 23 min at a flow rate of 400 µL/min. The 40 fractions were concatenated into 20, dried, and reconstituted in 3% acetonitrile and 0.1% trifluoroacetic acid.

### nanoLC-MS/MS analysis and database search

Each fraction was analyzed using an Orbitrap Lumos Tribrid mass spectrometer equipped with a FAIMS Pro ion mobility system interfaced with an nLC 1200 liquid chromatography system (all from Thermo Fisher Scientific). Peptides were trapped on an Acclaim Pepmap 100 C18 trap column (100 μm × 2 cm, 5 μm, Thermo Fischer Scientific) and separated on a custom-built analytical column (350 mm × 0.075 mm I.D.) packed with 3 μm Reprosil-Pur C18-AQ particles (Dr. Maisch, Ammerbuch-Entringen, Germany). Separation used a gradient from 3% to 80% acetonitrile in 0.2% formic acid over 85 min at a flow rate of 300 nL/min. FAIMS Pro switched between compensation voltages of -40 and -60, with nearly identical data-dependent settings at each voltage. Precursor ion mass spectra were acquired at 120,000 resolution, with a scan range of 450-1375 m/z, and a maximum injection time of 50 ms. MS2 analysis was performed in a data-dependent mode, where the most intense doubly or multiply charged precursors were isolated in the quadrupole with a 0.7 m/z window and dynamic exclusion within 10 ppm for 60 s. These precursors were fragmented by collision-dissociation at 35% collision energy, with a maximum injection time of 50 ms over 3 s (‘top speed’ setting). Fragment ions were detected in the ion trap, followed by multinotch (simultaneous) isolation of the top 10 MS2 fragment ions within an m/z range of 400-1400, with MS3 fragmentation by higher-energy collision dissociation at 65% collision energy. Detection of MS3 fragments was performed in the Orbitrap at 50,000 resolution, within an m/z range of 100-500, and a maximum injection time of 105 ms.

The data files for each set were combined for identification and relative quantification using Proteome Discoverer version 2.4 (Thermo Fisher Scientific). The search was performed against the *Homo sapiens* SwissProt database (March 2022) using Mascot 2.5 (Matrix Science) as the search engine, with a precursor mass tolerance of 5 ppm and a fragment mass tolerance of 0.6 Da. Tryptic peptides were accepted with zero missed cleavages, with variable modifications including methionine oxidation and fixed cysteine alkylation, along with TMT-label modifications at the N-terminus and lysine. Percolator was employed for PSM validation, applying a strict FDR threshold of 1%. TMT reporter ions were identified within a 3 mmu mass tolerance in the MS3 higher-energy collision dissociation spectra, and the TMT reporter abundance values for each sample were normalized based on the total peptide amount. Only quantitative results from unique peptide sequences with a minimum SPS match of 50% and an average signal-to-noise ratio (S/N) above 10 were used for protein quantification. The reference samples served as the denominator in the calculation of ratios. Quantified proteins were filtered at 1% FDR and grouped based on shared sequences to minimize redundancy.

### Synthetic bacterial vesicle (SyBV) preparation

SyBV were produced as previously described [21]. Briefly, E. coli bacterial cultures were pelleted, resuspended in 20 mM Tris-HCl (pH 8.0) in 20% sucrose, and treated with lysozyme (600 µg/g cells) and 0.1 M EDTA (0.2 mL/g cells) to produce spheroplasts. Then, the spheroplasts were pelleted and sonicated in 10 mM Tris-HCl (pH 8.0) at 4 °C using a Q55 Sonicator (20 kHz, QSonica, Newtown, CT) with the settings: amplitude 40, duration 2 min, and a 3 mm diameter ultrasound probe. Unbroken cells were removed by centrifugation at 8,000 x g for 5 min, and the supernatant was then spun at 40,000 x g for 1 h at 4 °C to collect whole membranes. The membranes were incubated in 0.5% Sarkosyl (Sigma Aldrich, St. Louis, MO, USA) for 20 min, after which the outer membranes were pelleted at 40,000 x g for 1 h at 4 °C. The pellets were incubated with a high pH solution (200 mM Na_2_CO_3_, pH 11) for 1 h at 25 °C, then loaded onto 4 mL of 50% iodixanol (Axis-Shield PoC AS, Oslo, Norway), followed by the addition of 4 mL of 30% iodixanol and 2 mL of 10% iodixanol in an ultracentrifuge tube. The layer formed between the 10% and 30% iodixanol after ultracentrifugation at 100,000 x g (28,000 rpm) in a SW 41 Ti Swinging-Bucket Rotor (Beckman Coulter, k-factor 145) for 2 h at 4 °C was collected. Finally, the samples were sonicated for 30 min using an ultrasonic bath (Grant, Cambridge, UK).

### Anti-tumor experimentation

Two mouse melanoma tissues were used to isolate EVs, using the same isolation process as for human melanoma tissues. C57BL/6 mice (6-8 weeks old, male) were subcutaneously inoculated in the right flank with B16F10 melanoma cells (5 × 10^5^) suspended in 100 µL of sterile PBS. Tumor growth was monitored daily until a measurable tumor mass (2-3 mm in diameter) formed, at which point mice were randomly assigned to experimental groups (*N* = 4 per group). Tumor EVs from either fresh or frozen mouse melanoma tissues and SyBV were prepared as described above. For treatment (Fig. 6A), EVs (5 × 10^5^) or SyBV (5 × 10^5^) were suspended in 100 µL PBS and subcutaneously injected near the established tumor mass. Based on our previous study, these vesicles were administered subcutaneously to maximize tumor targeting, as intravenous delivery resulted in substantial sequestration of vesicles in the liver and kidneys [22]. Injections were performed four times at three-day intervals. The sham control group received 100 µL of PBS at the same time points. Two independent batches of melanoma-derived EVs from both fresh and frozen tissues were used to ensure reproducibility, and EVs from each batch originated from the same melanoma tissue source. Consistent with our prior findings, EV- or SyBV-alone controls were omitted, as only the combination elicits tumor-specific adaptive immunity [21]. Tumor size was measured daily using digital calipers, and tumor volume was calculated using the formula (width)^2^ × (length) / 2. Mice were monitored for overall health and body weight throughout the study, and humane endpoints were applied in accordance with institutional animal care guidelines.

### Bioinformatics and statistical analysis

Where appropriate, the data in the graphs are reported as the mean ± standard deviation. The statistical analysis was performed using GraphPad Prism 6 (GraphPad Software, Inc., La Jolla, CA). For comparison between two groups, a paired Wilcoxon signed-rank test was used. Two-way ANOVA with two independent variables, followed by Tukey’s multiple comparison test, was used for multiple comparisons. The Shapiro-Wilk test was used to assess data normality.

To analyze the proteomic data, we used the Qlucore Omics Explorer software (Qlucore, Lund, Sweden) for the principal component analysis. We used the open-access analysis software Funrich [53] to compare the proteins listed in the Vesiclepedia [54] and ExoCarta [55] databases with our data.

## Results

### Human metastatic melanoma tissue analysis reveals a complex immune microenvironment

To contextualize the cellular source of EVs, we first determined the cellular composition of fresh human metastatic tissues harvested from either cutaneous melanoma or lymph node metastases using flow cytometry. The markers used were CD45 (white blood cells), CD3 (T cells), and SOX10 (melanoma cells) [56]. Firstly, we evaluated the expression of these markers in control samples (Additional file 2: Fig. S2). In PBMC, we gated for monocytes and lymphocytes, which were CD45^+^ and CD45^+^CD3^+^, respectively. Importantly, they were both negative for SOX10 (Additional file 2: Fig. S2A). The melanoma cell line, MML-1, was negative for CD45 and CD3, but positive for SOX10 (Additional file 2: Fig. S2B). Lastly, we also evaluated the liver cancer cell line, HepG2, which was not positive for any of the markers (Additional file 2: Fig. S2C). Together, these results show that CD45 and CD3 can be used to identify immune cells, while SOX10 can be used to identify melanoma cells.

**Fig. 2:**
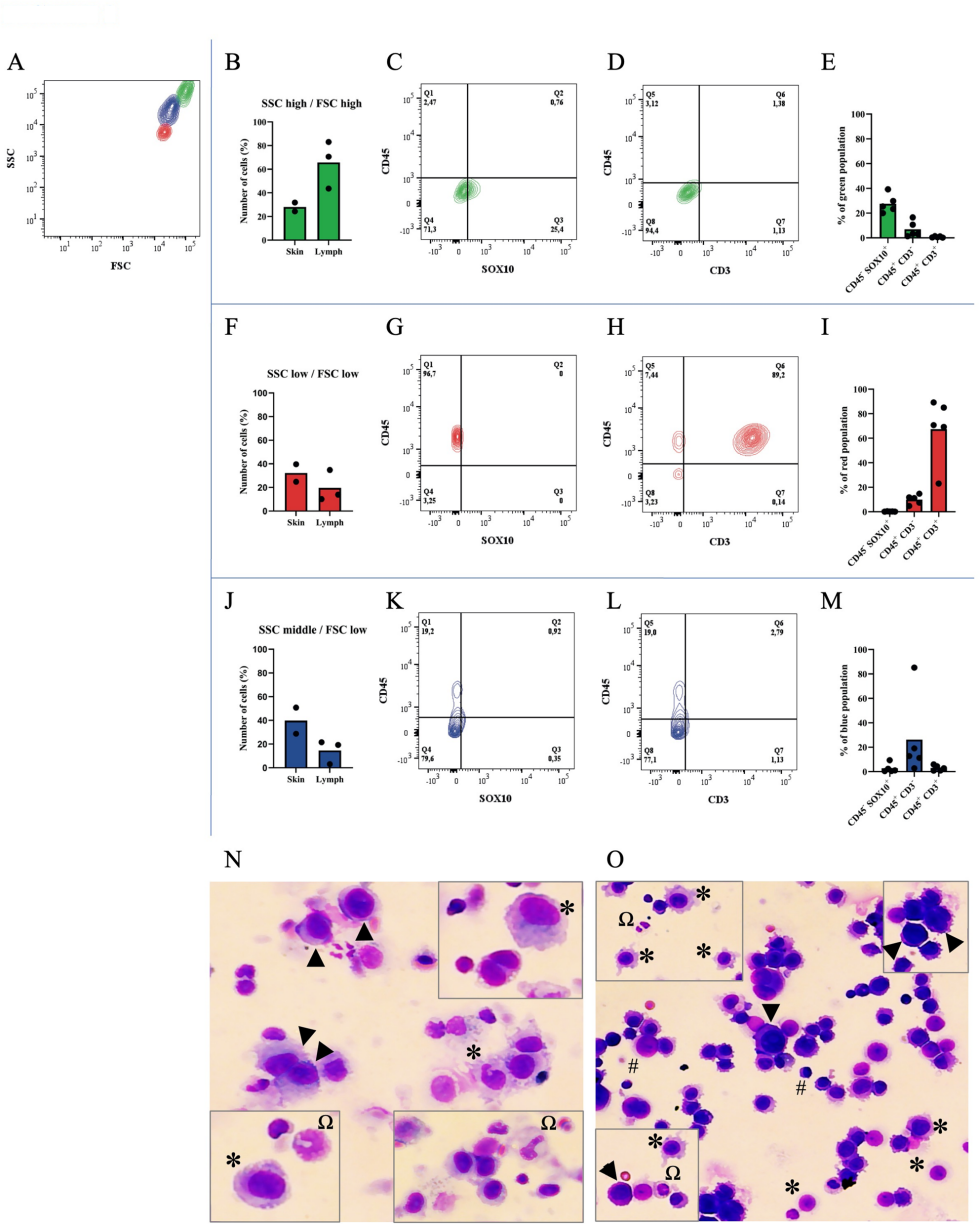
Cellular composition of human melanoma metastatic tissues. **A** Flow cytometry analysis of fresh human melanoma metastases identified three distinct cell populations based on SSC-A and FSC-A parameters: a population of large, highly granular cells (SSC^high^/FSC^high^, green), a small, low-granularity population (SSC^low^/FSC^low^, red), and an intermediate population (SSC^middle^/FSC^low^, blue). **B**, **F**, **J** Bar graphs show the proportion of each population within skin and lymph node metastases. **C**, **G**, **K** Representative dot plots showing CD45 and SOX10 expression within the green, red, and blue cell populations, respectively. **D**, **H**, **L** Representative dot plots showing CD45 and CD3 expression within each of the three populations. **E**, **I**, **M** Quantification of the percentage of cells within each population expressing CD45^+^/SOX10^+^, CD45^+^/CD3^–^, and CD45^+^/CD3^+^ phenotypes. *N* = 5. Representative pictures of Hemacolor Rapid staining of the single cell-suspension of **N** human melanoma skin metastasis and **O** human lymph node metastasis. Morphological analysis was performed to identify the cell types. Black arrows indicate tumor cells; * indicates macrophages; Ω indicates neutrophils; and # indicates lymphocytes. *N* = 5

In all the tumoral tissues, three distinct cell populations were identified by FACS using size and granulation gating (Fig. 2A). Live cells (aquaDL-negative) were then further evaluated for CD45/SOX10 and CD45/CD3. The results showed that most of the cells had high side scatter (SSC) and forward scatter (FSC) values (30-80%, hereafter marked in green), indicating that they were large and with high intracellular complexity (Fig. 2B). Moreover, they were negative for CD45 and CD3 but positive for SOX10 (Fig. 2C-E), suggesting that this subpopulation of cells was mainly composed by tumor cells. The other two subpopulations of cells, which were smaller and less granulated, were present at similar percentages in both tissue types (10-40%). One of these cell populations (hereafter marked in red) was identified as T-lymphocytes based on positivity for CD45 and CD3, accounting for approximately 30% in the skin metastases and 20% in the lymph node metastases (Fig. 2F-I). A mixture of different non-lymphatic cell types constituted the third subpopulation (hereafter marked in blue), which had a substantially lower expression of the three assessed markers (Fig. 2J-M). The CD45^–^ and SOX10^–^ populations likely represented other cells of the tumor microenvironment, such as cancer-associated fibroblasts. Overall, the observed representation of the different cell populations was in line with our previous study [23] and was confirmed by morphological analysis using Hemacolor Rapid staining of cytospin samples. As expected, in the skin metastatic samples, we detected tumor cells, macrophages, and neutrophils (Fig. 2N), while in the lymph node metastatic tissues, a large number of lymphocytic-like cells were identified, along with macrophages, neutrophils, and tumor cells (Fig. 2O). These results highlight the complex immune-tumor microenvironment within clinically relevant human melanoma metastatic tissues.

### Cryopreservation does not introduce systematic changes in EV profiles

**Fig. 3:**
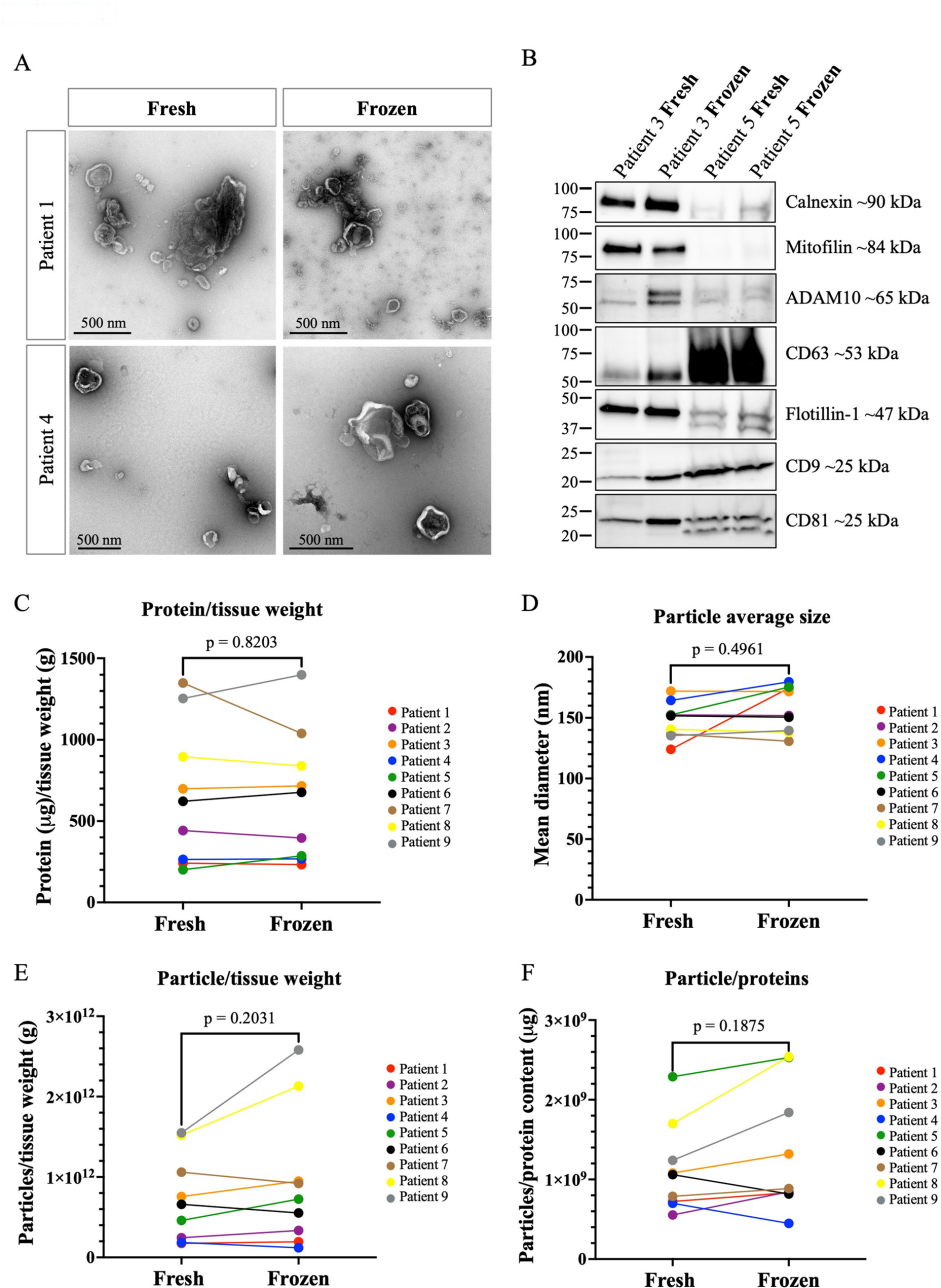
Characterization of the EVs isolated from the fresh and frozen human melanoma tissues. **A** Representative microphotographs of EVs isolated from fresh and frozen human melanoma tissues. Scale bar: 500 nm. *N* = 2. **B** Western blot performed on both experimental groups from two patients to evaluate the presence of EV markers such as CD9, CD63, CD81, and Flotillin-1 as well as Mitofilin, ADAM10, and Calnexin, an ER-derived protein. *N* = 2. The similarity between EVs from the fresh and frozen tissue samples was assessed and compared in terms of **C** protein/tissue weight ratio, **D** particle average size, **E** particles/tissue weight ratio, and **F** particles/protein ratio. A paired Wilcoxon signed-rank test was used to compare two groups. *N* = 9

After the evaluation of the tissue cellular composition, we isolated and characterized EVs from both fresh and frozen human melanoma metastatic tissues as described in Fig. 1. The TEM images showed that EVs from both fresh and frozen tissues exhibited the typical EV morphology and size (30-500 nm) (Fig. 3A, and higher magnification in Additional file 5: Fig. S5). Typical EV markers and potential contaminants were also investigated in the two EV preparations, as suggested by the MISEV guidelines [57, 58]. Classic EV markers like CD9, CD63, CD81, and Flotillin-1 were detected in EVs isolated from both fresh and frozen tissues of the two patients, while Mitofilin was only positive in EVs isolated from patient 3 (Fig. 3B, full-length blots are presented in Additional File 2: Fig. S2). In addition, the presence of ADAM10, previously reported to be enriched in melanoma-derived small EVs compared to large EVs [23], was detected in all of the analyzed samples. Calnexin, an endoplasmic reticulum marker, was detected in both fresh- and frozen-derived EVs, with higher expression in EVs from patient 3 and minimal levels in those from patient 5. Importantly, even if EVs isolated from two different human patients did not express the investigated markers at the same levels, the signals for most of the markers showed comparable trends in terms of presence and strength between EVs from fresh and frozen tissue collected from the same patient. As the observed inter-patient variability exceeded intra-group variability, these results indicate that cryopreservation did not introduce systematic changes in EV morphology and classic EV marker expression.

We next evaluated the yield and the purity of the EV preparations from both fresh and frozen tissues. Therefore, we first quantified the protein concentration in the two experimental groups, normalizing the data to the weight of the tissue collected from each patient. This ratio was comparable in the EVs isolated from the fresh and the frozen human tissues in all the 9 patients of this study (Fig. 3C). Similarly, the average particle diameter was not significantly different between fresh and frozen samples within the same patient (Fig. 3D and Additional file 6: Fig. S6). We also analyzed the ratio between the number of particles and the weight of the tissue. As shown in Fig. 3E, the ratio was slightly higher in EVs isolated in the frozen samples of 5 out of 9 patients compared to those obtained from the fresh ones, indicating that a slightly higher number of nanosized particles was present in these specimens. Importantly, no statistically significant differences were observed comparing fresh and frozen samples. Lastly, to evaluate the purity of EV preparations, we calculated the ratio between the particle number and the protein concentration. A similar trend emerged for this ratio: in most patients (7 out of 9), the ratio was slightly higher in the frozen group, except for patients 4 and 6, who showed the opposite trend. Overall, no significant differences were evident between the two experimental groups in all the patients (Fig. 3F). These findings confirm that EVs from cryopreserved tissue retain structural integrity and biochemical consistency with their fresh counterparts.

### Quantitative proteomics confirms stability of EV profiles post-freezing

Next, we performed quantitative TMT-based proteomic analysis of EVs isolated from fresh and frozen human tissues to further characterize EV composition and, using high-resolution mass spectrometry, assess potential cell-derived contamination introduced during freezing. A total of 8,251 proteins were identified, and of these, 7,573 were quantified (all proteins are listed in Additional file 14: Table S5).

**Fig. 4:**
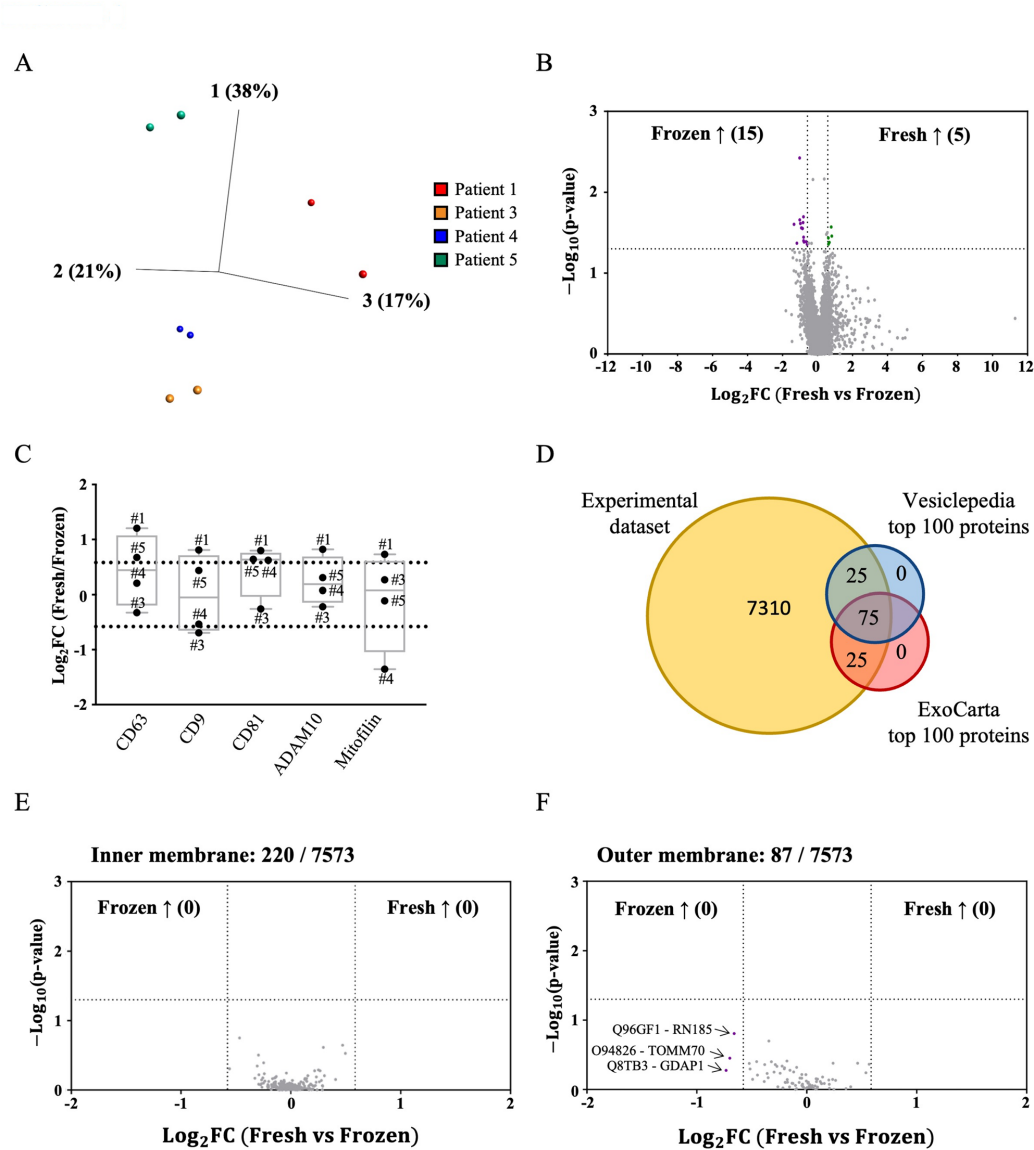
Quantitative proteomic analysis (TMT) performed on the fresh and frozen tissue-derived EVs. **A** PCA illustrating the relation between EVs isolated from different patients. **B** The volcano plot shows the 15 and 5 proteins that were significantly enriched in frozen (purple) and in fresh (green) tissue-derived EVs, respectively. *N* = 4. **C **Fold change values of EV markers (CD63, CD9, CD81, ADAM10, Mitofilin) in fresh versus frozen samples. *N* = 4. **D** Venn diagram showing the comparison of the whole protein dataset obtained from the four patients (yellow) and the Vesiclepedia (blue) (54) and ExoCarta (red) (55) databases. Volcano plots of the inner (**E**) and outer (**F**) membrane mitochondrial proteins found in the experimental dataset. Grey dotted lines show p-value < 0.05- and 1.5-fold change cut-offs

First, we performed a principal component analysis (PCA) on the quantified proteins to visualize the relation between the different groups of EV samples. The PCA plot revealed that EVs clustered primarily by patient rather than by tissue condition (fresh or frozen), indicating that intra-patient similarity exceeded any differences potentially introduced by cryopreservation (Fig. 4A). The same plot is shown in Additional file 3: Fig. S3, but with coloring based on fresh vs. frozen tissue-derived EVs instead of coloring based on patients. These results indicate that intra-group variability of the protein cargo was lower than inter-patient variability, confirming that cryopreservation does not substantially alter the EV proteome composition.

Very small differences in protein content in fresh and cryopreserved human tissue-derived EVs were observed by volcano plot analysis (Fig. 4B). Comparing the normalized protein abundance between fresh and frozen samples, only 15 proteins, representing the 0.2% of the total quantified proteins, were significantly enriched in the frozen samples, while even fewer (5 proteins) were statistically enriched in the fresh samples (listed in Additional file 10: Table S1). Statistical enrichment is defined as a statistically significant difference in protein abundance between fresh and frozen conditions (p-value < 0.05 and fold change ≥ 1.5). In addition, only 8 proteins showed a fold change greater than 16 in the fresh sample compared to the frozen sample (Fig. 4B, right bottom corner of the plot), although none of them resulted statistically significant (Additional file 13: Table S4). Altogether, these results confirmed the similarity of the whole proteome in the two experimental groups.

To further determine whether the frozen-derived EVs maintain the same characteristics as the fresh counterpart, we compared the relative abundance of five commonly EV-enriched proteins (CD63, CD9, CD81, ADAM10, and Mitofilin) [23, 59], which were also previously assessed by Western blot. The log fold changes of these five proteins ranged between 1.2 and 1.3, indicating no major difference in their abundance in isolates from fresh or frozen tissues (Fig. 4C). While such values correspond to approximately 2.3- to 2.5-fold differences and can be biologically relevant in specific contexts, in this particular case, these variations did not reach statistical significance and were not consistently observed across all samples. In fact, these changes emerged only for CD63 and Mitofilin, each occurring in a single patient and in opposite directions. In addition, our dataset included the top 100 most-represented proteins from the Vesiclepedia [54] (http://microvesicles.org/) and ExoCarta [55] (http://exocarta.org/index.html) databases, of which 75 were common to both (Fig. 4D). This comparison showed that most of the proteins identified in our samples are associated with EVs, as reported in previous studies. This observation confirmed not only the identity of frozen tissue-derived EVs, but also their close resemblance to those derived from fresh tissue.

To assess the potential presence of intracellular components in human tissue-derived EVs resulting from cell disruption during freezing, we focused on mitochondrial proteins as potential markers of such contamination. We identified 220 proteins associated with the mitochondrial inner membrane according to UniProt, and none of them showed a significant enrichment in the frozen samples compared to the fresh samples (Fig. 4E). Among the 87 mitochondrial outer membrane proteins that were detected, three showed a small and non-significant enrichment in the frozen samples (Fig. 4F and Additional file 12: Table S3).

Overall, the proteomic profiling results demonstrated that the protein cargo of frozen tissue-derived EVs was comparable to that of EVs isolated from fresh tissues, confirming that cryopreservation does not significantly affect the composition and purity of tumor-derived EVs, thereby supporting their potential use in clinical diagnostic pipelines.

### Diagnostic markers remain detectable in cryopreserved tissues

**Fig. 5:**
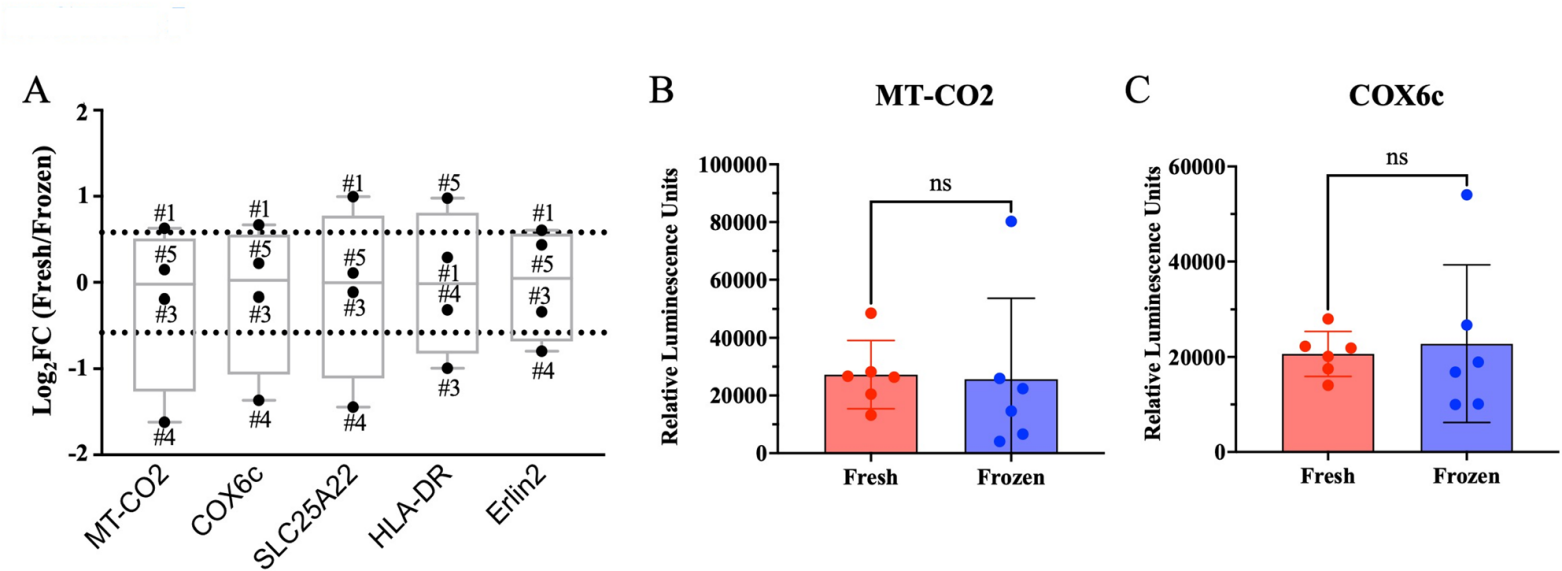
Evaluation of the potential diagnostic value of fresh and frozen tissue-derived EVs. **A** The relative quantity of five potential EV-melanoma markers (MT-CO2, COX6c, SLC25A22, HLA-DR and Erlin2) was determined from the proteomic analysis performed on the two experimental groups. The data are reported as the average fold change in the ratio between the fresh and frozen tissue-derived EVs. *N* = 4. ELISA assay of (**B**) MT-CO2 and (**C**) COX6c levels obtained by comparing all fresh versus all frozen EV samples collectively. Data are presented as mean ± SEM. A paired Wilcoxon signed-rank test was used to compare two groups. ns = not significant, *N* = 6

To assess diagnostic value, we aimed to determine whether EVs isolated from cryopreserved human tissue retained their suitability as biomarkers. To this aim, we compared the relative abundances from mass spectrometry analysis of five proteins (MT-CO2, COX6c, SLC25A22, HLA-DR, and Erlin2) that we previously showed to be enriched in EVs isolated from fresh human melanoma tissue [19]. As for the EV-specific markers, some inter-patient variability emerged from this analysis. In particular, three of these five proteins (MT-CO2, COX6c, SLC25A22) were slightly increased in the fresh samples of patient 4. Despite this variability, no significant differences in the levels of these five proteins emerged between fresh and frozen tissue-derived EVs (Fig. 5A). Similar outcomes emerged from the ELISA assays performed to validate the results of the two markers (MT-CO2 and COX6c) previously shown to be enriched in the plasma of patients affected by melanoma, breast cancer, and ovarian cancer [19]. No statistically significant differences were detected between fresh versus frozen EV samples (Fig. 5B-C), although slight variations between fresh and frozen tissue-derived EVs from the same patient were observed (Additional file 9: Fig. S9A, B). These findings were further supported by Western blot analyses, which similarly showed no significant differences between the two conditions (Additional file 9: Fig. S7C, D; full-length blots are presented in Additional File 7: Fig. S7A, B). These findings suggest that the EVs isolated from frozen tissues retain potential clinically relevant biomarker expression, making them suitable for translational applications in cancer detection and monitoring.

### EVs from cryopreserved melanoma tissues maintain antitumor function in vivo

Finally, we compared the immunotherapeutic capability of EVs derived from fresh and frozen tissues. Firstly, we isolated EVs from fresh and frozen mouse melanoma tissues using the same approach previously described for the human samples. Characterization of these EVs further demonstrated the similarity of EVs isolated from fresh and cryopreserved tissues. In fact, no significant differences were observed in EV morphology (Additional file 8: Fig. S8A), nor in EV preparation parameters, including the particle size distribution (Additional file 8: Fig. S8B, C), protein/tissue weight ratio (Additional file 8: Fig. S8D), particle/tissue weight ratio (Additional file 8: Fig. S8E), and particle/protein ratio (Additional file 9: Fig. S9F).

**Fig. 6:**
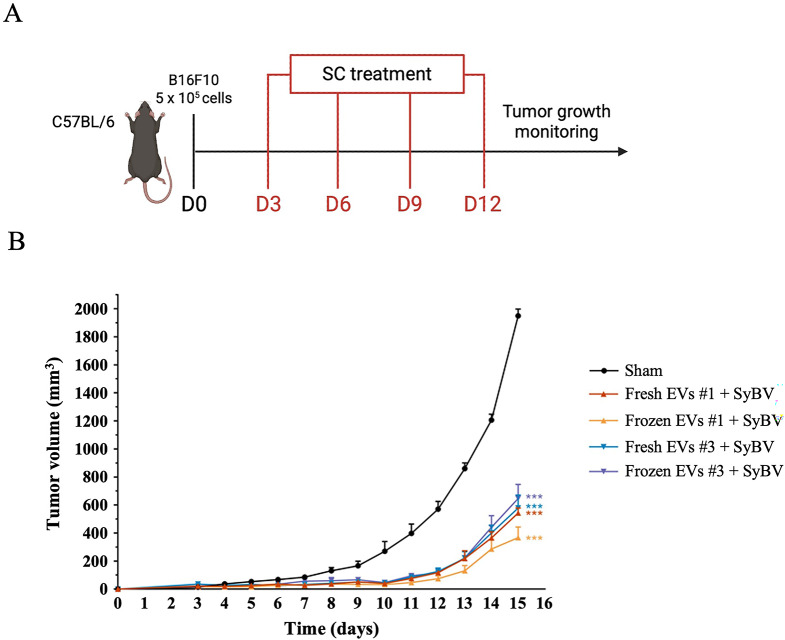
Comparison of the immunotherapeutic potential of fresh and frozen murine tissue-derived EVs in melanoma-bearing mice.** A** Graphical representation of the in vivo experimental design: mice were inoculated with 5 × 10^9^ tumor-derived EVs (from either fresh or frozen tissues) and 5 × 10^9^ SyBV four times at 3-day intervals following B16F10 inoculation. **B** Tumor growth curve of mice treated with fresh or frozen mouse-derived EVs combined with SyBV (*N* = 4). Data are present as mean ± SEM *** = *p* < 0.001 by one-way-ANOVA with Turkey´s post test versus sham group. *N* = 4

Then, to compare the immunotherapeutic potential of fresh and frozen tissue-derived EVs, we analyzed the tumor growth in an immunized mouse model. Briefly, tumoral B16F10 cells were subcutaneously (SC) established in the flanks of mice. Once the tumor mass reached palpable size, mice were SC injected four times at 3-day intervals with both fresh and frozen mouse melanoma-derived EVs, together with SyBV (Fig. 6A). The results revealed that the mice treated with the combination of tumor-derived EVs from both fresh and frozen tissues and SyBV showed significantly reduced tumor growth compared to control mice. The injection of frozen tissue-derived EVs and SyBV resulted in a similar reduction of tumor volume observed with fresh-derived EVs, with no statistical difference between the two groups (74% vs. 71.2%) (Fig. 6B). These findings demonstrate that frozen tissue-derived EVs retain the same functionality of their fresh counterparts and underline the potential of using cryopreserved tumor tissues to develop EV-based cancer vaccines or immunotherapeutic formulations, bridging basic research with clinical translation.

## Discussion

In this study, we showed that EVs isolated from cryopreserved melanoma tissues have morphological and compositional features similar to those of EVs from fresh tissues. Furthermore, proteomic analysis revealed a 99% overlap between EVs from fresh and frozen tissues, confirming that cryopreservation maintains overall tissue-EV integrity. Consistent with this, the levels of previously identified cancer-associated EV biomarkers, including MT-CO2 and COX6c [19], were comparable between the two preparations. Finally, we showed that EVs derived from cryopreserved tissues significantly inhibited tumor progression in vivo, with antitumor effects comparable to those of EVs derived from fresh tissues.

Our findings align with recent work by Shen et al., who reported preservation of EV characteristics in frozen ovarian cancer tissues [29]. While their study primarily focused on structural and molecular characterization, our work extends these findings by investigating a different tumor context, evaluating potential disease-associated biomarkers, and incorporating functional in vivo validation. Together, these studies support the emerging idea that cryopreserved tissues could be a viable source of EVs.

A key finding supporting the translational use of frozen tissue-derived EVs is their strong structural and molecular consistency with EVs from fresh melanoma tissues. TEM micrographs from both human and mouse samples showed no structural differences or increased debris after freezing. Proteomic analysis confirmed this similarity, showing no significant protein enrichment in either condition. Only 15 of 7,573 proteins were slightly enriched in frozen-derived EVs, all intracellular and representing ~ 0.2% of the total proteome, indicating minimal contamination induced by the freezing process.

To isolate EVs from both fresh and frozen tissues, we employed a well-established, widely recognized protocol to obtain high-purity preparations. However, as specified in the MISEV guidelines [60], the complete elimination of non-vesicular contaminants is not technically achievable; instead, they recommend defining EV preparations based on the enrichment of characteristic EV-associated markers. Our results align with prior studies demonstrating that, even in highly optimized preparations, small amounts of non-vesicular proteins, such as lipoproteins or soluble plasma proteins, are invariably co-isolated [61–63]. Keeping in mind the limit of any EV-isolation protocols, including ours, it is important to underline that the aim of the present study was not to optimize EV isolation protocols for maximal EV purity, but rather to compare EVs obtained from fresh and frozen tissues in order to evaluate the feasibility of using frozen samples as a reliable source of EVs. Within this context, the minimal differences observed between conditions support the suitability of frozen tissues for EV-based analyses.

The human melanoma metastases analyzed in this study were collected from skin, lymph node, and intestine, sites with distinct tissue architectures. Despite these differences, EVs isolated from all locations showed consistent morphology, yield, and proteomic profiles, demonstrating the robustness of the used isolation workflow across diverse tumor microenvironments. Although our findings are specific to melanoma, a strongly immunogenic tumor with well-characterized biomarkers [23, 59], the approach can be readily extended to other cancer types. As a proof-of-concept study, we analyzed nine patient-derived melanoma tissue samples, acknowledging that the relatively small cohort size, sex imbalance, and lack of healthy control tissues reflect the challenges with obtaining metastatic specimens in a clinical setting. Future studies involving larger, more diverse patient cohorts spanning different ages, genders, disease stages, and melanoma subtypes will be essential to fully establish the clinical applicability of this strategy.

From a technical perspective, our iodixanol-based EV purification protocol yielded highly reproducible results and is well suited for biomarker discovery applications [19, 64]. However, the procedure is time-consuming and requires specialized expertise as well as access to advanced instrumentation. Future efforts should therefore focus on streamlining and miniaturizing EV isolation workflows to facilitate their broader implementation in translational research and biomarker discovery pipelines.

We evaluated the effects of a single, albeit the most commonly used, tissue cryopreservation method for EV characterization, namely snap freezing. This approach is generally considered less damaging than slow freezing [65]. In addition to the freezing process, recent studies indicate that the thawing step is also critical for preserving the structural, biochemical, and functional integrity of EV preparations [50, 51]. In accordance with MISEV2018 guidelines, we used slow thawing on ice, which is considered superior for EVs [50].

Moreover, the results reported in this work are obtained from tissues stored at − 80 °C for a relatively short period (two weeks), which does not fully recapitulate the long-term storage conditions typical of biobanked specimens. While EV morphology, protein composition, and functional properties were well preserved under these conditions, the impact of longer-term storage remains to be determined. It is important to underline that the aim of the current study was to evaluate the effect of the freezing process itself on frozen tissue-derived EVs, rather than to model long-term biobanking. In this context, the approach, based on short-term cryopreservation, enabled a controlled, paired experimental design using matched fresh and frozen tissues from the same patient, thereby minimizing biological variability and allowing a direct assessment of freezing-induced alterations. Our findings demonstrate that the initial freezing step, an essential part of any biobanking workflow, does not compromise the structural integrity, molecular composition, or biological activity of EVs. This represents a critical prerequisite for the use of long-term biobanked tissues in EV-based translational applications. Building on the foundations, further studies are required to systematically investigate the effects of extended storage periods and alternative freezing strategies.

Within the marker panel analyzed by Western blot, we included Calnexin, an endoplasmic reticulum protein commonly used as an indicator of potential contamination from intracellular compartments. The detection of Calnexin in a subset of samples may reflect, at least in part, variable degrees of tissue degradation and the consequent release of intracellular components during tissue processing and EV isolation. However, increasing evidence indicates that Calnexin can be detected in EV preparations derived not only from melanoma but also from other cancerous and non-cancerous tissues [29, 42, 66–70], whereas it is absent in EVs isolated from human colon cancer and healthy mucosa processed using the same protocol as in this paper [71]. The observed variability suggests that its presence does not necessarily indicate impurity in EV isolates, while it may also reflect the biological complexity and cellular heterogeneity inherent to tissue-derived EV populations. Consistent with this variability, in the current study, Calnexin was detected in only one of the two EV samples analyzed by Western blot and was barely visible in the other. Notably, the observed variability was not restricted to Calnexin but was also evident in canonical EV markers, further supporting the heterogeneous nature of tissue-derived EVs. Importantly, the comparable levels observed between matched fresh and frozen samples indicate that cryopreservation per se does not exacerbate this potential contamination, supporting the validity of our comparative approach. The observation that cryopreservation was not associated with increased intracellular-derived contamination of EVs was further supported by analysis of mitochondrial proteins, which are often used as indicators of freeze-induced damage [72]. Mitochondrial proteins were detected at comparable levels in EVs from fresh and cryopreserved tissues, indicating that freezing did not alter EV protein composition. Importantly, the presence of mitochondrial proteins in EVs is not surprising, as they can be physiologically incorporated into EVs [73, 74] and have even been proposed as cancer EV biomarkers [19].

Although our data indicate that the freezing step does not significantly affect the abundance of selected EV-associated proteins (e.g., MTCO-2, COX6c, and EV-associated markers), the potential impact of freeze-thaw on protein conformation and epitope integrity should be carefully considered. It has been reported that freezing and thawing can induce structural alterations in proteins, potentially affecting epitope accessibility and, consequently, the performance of antigen presentation and antibody recognition processes [75, 76]. Therefore, while our findings support the feasibility of using frozen tissues as a source of structurally intact and functionally active EVs, additional validation will be important for specific translational or diagnostic applications.

Beyond their diagnostic value, our in vivo data highlight the functional relevance of tissue-derived EVs as immunotherapeutic agents. We previously showed that tissue-derived EVs combined with the SyBV adjuvant induce tumor-specific immune responses and reduce melanoma growth in melanoma-bearing mice [21]. Here, we demonstrated that EVs derived from frozen melanoma tissues retain antitumor activity and perform equivalently to those derived from fresh tissues. The in vivo experiment was intentionally short (16 days), as tumors reached excessive size by 18–20 days, exceeding ethical limits. Importantly, the primary objective of this experiment was to assess whether cryopreservation affects the immunogenic and antitumor properties of tissue-derived EVs, rather than to evaluate long-term therapeutic efficacy or survival. However, the in vivo assessment of the functional capacity of EVs derived from frozen vs. fresh tissues was primarily limited to tumor growth measurements. Although comparable anti-tumor effects were observed between fresh and frozen tissue-derived EVs, further studies will be required to fully define the therapeutic potential of cryopreserved EVs.

## Conclusions

These results support the potential use of EVs isolated from cryopreserved tissues for biomarker discovery and immuno-oncology studies. The preservation of immunological activity further suggests that such EVs may retain functional relevance in immuno-oncology applications. Given the practical challenges of collecting and immediately processing fresh tissues, our findings indicate that stored samples may be a feasible alternative source for EV-based analyses. This approach aligns with precision medicine initiatives and expands access to patient-derived material for translational research.

## Supplementary Information

Below is the link to the electronic supplementary material.

Supplementary Material 1: ADDITIONAL FILE 1: FIG. S1 FMO strategy used to define gating for SOX10, CD45, and CD3 across melanoma tissue–derived cell populations.

Supplementary Material 2: ADDITIONAL FILE 2: FIG. S2 Reference cell populations used to validate gating for SOX10, CD45, and CD3

Supplementary Material 3: ADDITIONAL FILE 3: FIG. S3 Representative close-up electron micrographs of EVs isolated from two patients from both fresh and frozen tissues.

Supplementary Material 4: ADDITIONAL FILE 4: FIG. S4 Uncropped Western Blots Full-length, uncropped Western Blots images corresponding to all cropped blots presented in Figure 3B.

Supplementary Material 5: ADDITIONAL FILE 5: FIG. S5 Size distribution of EVs isolated from each patient obtained using nanoparticle tracking analysis

Supplementary Material 6: ADDITIONAL FILE 6: FIG S6 PCA illustrating the relation between fresh (red) and frozen (blue) tissue-derived EVs

Supplementary Material 7:ADDITIONAL FILE 7: FIG. S7 MT-CO2 and COX6c expression in fresh and frozen EVs

Supplementary Material 8: ADDITIONAL FILE 8: FIG. S8 Uncropped Western Blots. Full-length, uncropped Western Blots images corresponding to allcropped blots presented in ADDITIONAL FILE 7: Fig. S7C and D

Supplementary Material 9: ADDITIONAL FILE 9: FIG. S9 Characterization of the EVs isolated from the fresh and frozen mouse melanoma tissues.

Supplementary Material 10: ADDITIONAL FILE 10: TABLE S1 Description of the 11-plex Tandem Mass Tag (TMT) labeling

Supplementary Material 11: ADDITIONAL FILE 11: TABLE S2 List of proteins quantified in EVs isolated from fresh and frozen human tissues. N = 4

Supplementary Material 12: ADDITIONAL FILE 12: TABLE S3 Significant proteins identified and quantified in EVs from fresh versus frozen tissues

Supplementary Material 13: ADDITIONAL FILE 13: TABLE S4 List of the 8 proteins enriched in EVs from fresh versus frozen tissues

Supplementary Material 14: ADDITIONAL FILE 14: TABLE S5 List of the 3 mitochondrial proteins enriched in EVs from frozen versus fresh tissues

## Data Availability

The proteomic datasets generated and analyzed during the current study are available in the ProteomeXchange Consortium via the PRIDE (77) repository with the identifier PXD068552.All relevant data of experiments have been submitted to the EV-TRACK knowledgebase (EV-TRACK ID: EV240018) (78).Other data and materials supporting the findings of this study are available from the corresponding author upon reasonable request.

## Abbreviations

ADAM10: A Disintegrin and Metalloproteinase Domain-Containing Protein 10
APC: Allophycocyanin
BCA: Bicinchoninic Acid
BSA: Bovine Serum Albumin
bRP-LC: Basic Reversed-Phase Liquid Chromatography
CD: Cluster of Differentiation
CCD: Charge-Coupled Device
CO_2_: Carbon Dioxide
COX6c: Cytochrome c Oxidase Subunit 6 C
DMEM: Dulbecco’s Modified Eagle Medium
DNase: Deoxyribonuclease
DTT: Dithiothreitol
EBM: Experimental Biomedicine
EDTA: Ethylenediaminetetraacetic Acid
ELISA: Enzyme-Linked Immunosorbent Assay
ER: Endoplasmic Reticulum
EV(s): Extracellular Vesicle(s)
EV-TRACK: Extracellular Vesicle Transparent Reporting and Centralizing Knowledge
FAIMS: Field Asymmetric Ion Mobility Spectrometry
FACS: Fluorescence-Activated Cell Sorting
FASP: Filter-Aided Sample Preparation
FBS: Fetal Bovine Serum
FDR: False Discovery Rate
FMO: Fluorescence Minus One
FSC: Forward Scatter
GPC-1: Glypican-1
HCD: Higher-Energy Collision Dissociation
HEK293: Human Embryonic Kidney 293 cells
HepG2: Human Hepatocellular Carcinoma Cell Line
HLA-DR: Human Leukocyte Antigen - DR isotype
HRP: Horseradish Peroxidase
LC-MS/MS: Liquid Chromatography Tandem Mass Spectrometry
L-EVs: Large Extracellular Vesicles
MISEV: Minimal Information for Studies of Extracellular Vesicles
miRNA: MicroRNA
MT-CO2: Mitochondrially Encoded Cytochrome c Oxidase II
NTA: Nanoparticle Tracking Analysis
PBMC: Peripheral Blood Mononuclear Cells
PBS: Phosphate-Buffered Saline
PCA: Principal Component Analysis
PD-L1: Programmed Death-Ligand 1
PerCP: Peridinin-Chlorophyll Protein
PSM: Peptide-Spectrum Match
PVDF: Polyvinylidene Difluoride
RIPA: Radioimmunoprecipitation Assay Buffer
RPMI: Roswell Park Memorial Institute Medium
RT: Room Temperature
SDS-PAGE: Sodium Dodecyl Sulfate Polyacrylamide Gel Electrophoresis
SOX10: SRY-Box Transcription Factor 10
SSC: Side Scatter
SyBV: Synthetic Bacterial Vesicles
TEAB: Triethylammonium Bicarbonate
TEM: Transmission Electron Microscopy
TFA: Trifluoroacetic Acid
TMT: Tandem Mass Tag
TNM: Tumor-Node-Metastasis
UPLC: Ultra-Performance Liquid Chromatography
WB: Western Blot
