# Supplementary figures and images for: Frozen melanoma tissues yield extracellular vesicles with preserved diagnostic and immunogenic properties
Source: BMC Med. 2026 May 21;24:325. doi: 10.1186/s12916-026-04923-8 (PMC13198046; doi:10.1186/s12916-026-04923-8)

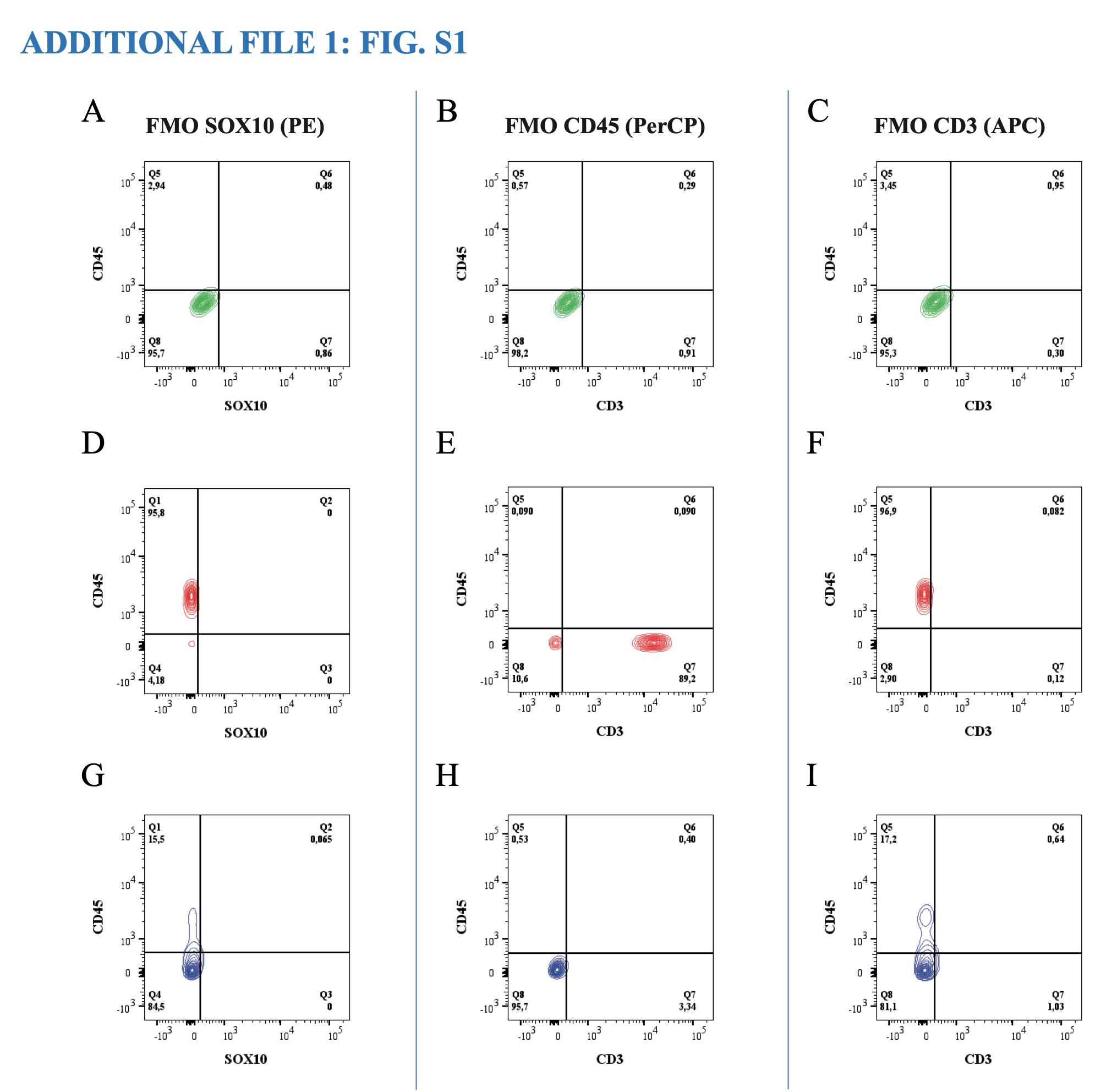

Supplement: Supplementary file 1 — Supplementary Material 1: ADDITIONAL FILE 1: FIG. S1 FMO strategy used to define gating for SOX10, CD45, and CD3 across melanoma tissue–derived cell populations. [file 12916_2026_4923_MOESM1_ESM.jpg]

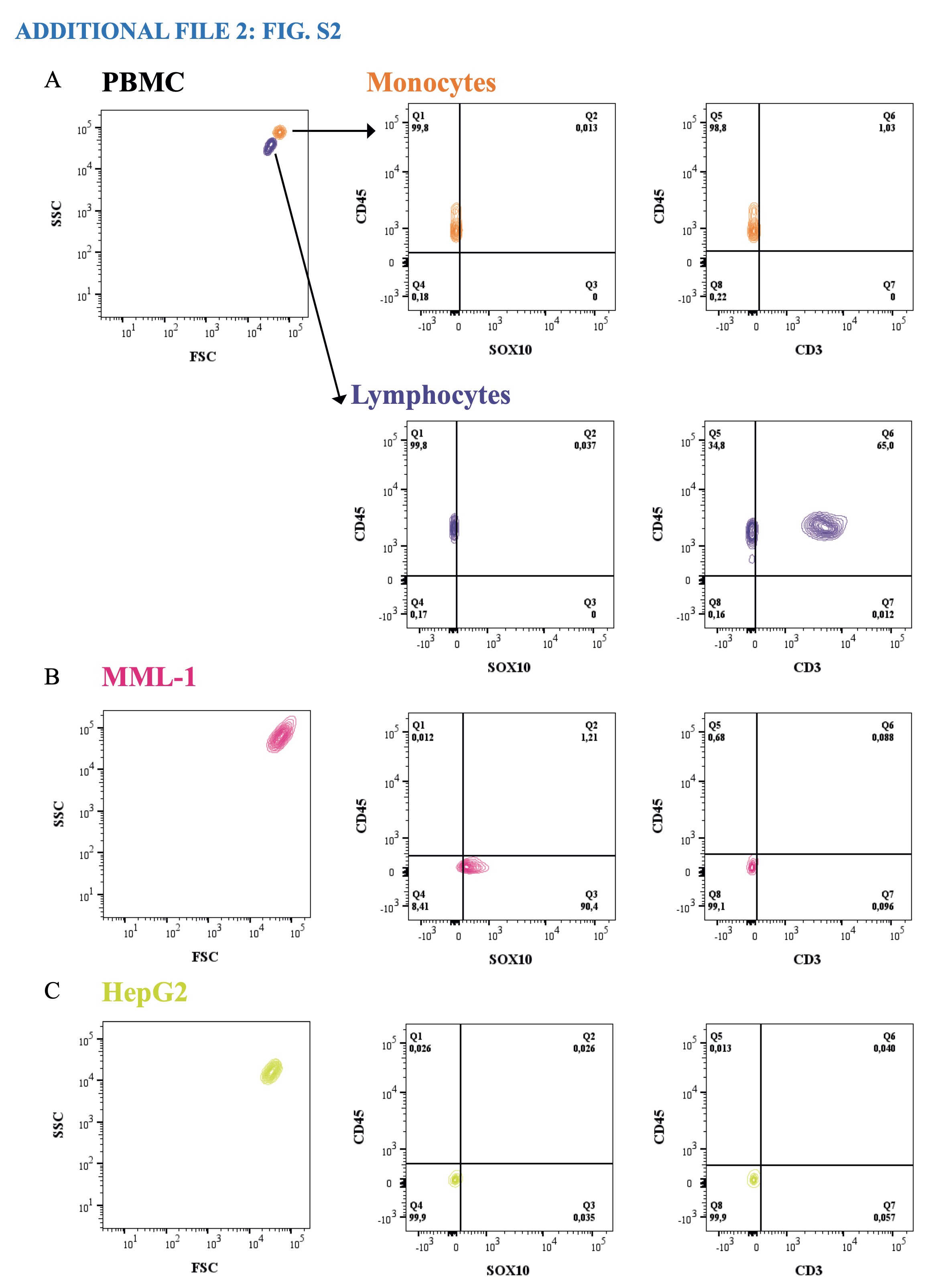

Supplement: Supplementary file 2 — Supplementary Material 2: ADDITIONAL FILE 2: FIG. S2 Reference cell populations used to validate gating for SOX10, CD45, and CD3 [file 12916_2026_4923_MOESM2_ESM.jpg]

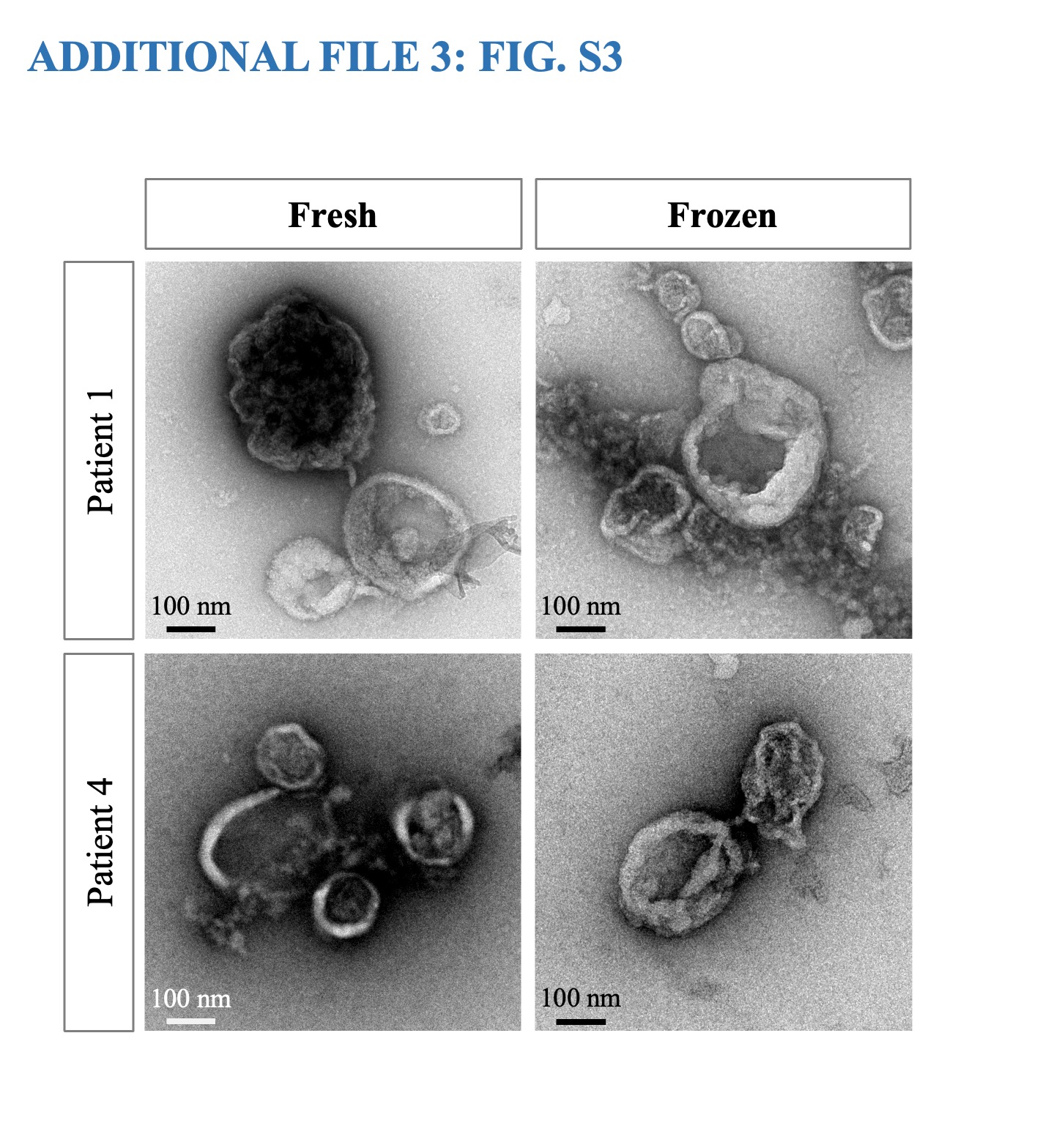

Supplement: Supplementary file 3 — Supplementary Material 3: ADDITIONAL FILE 3: FIG. S3 Representative close-up electron micrographs of EVs isolated from two patients from both fresh and frozen tissues. [file 12916_2026_4923_MOESM3_ESM.jpg]

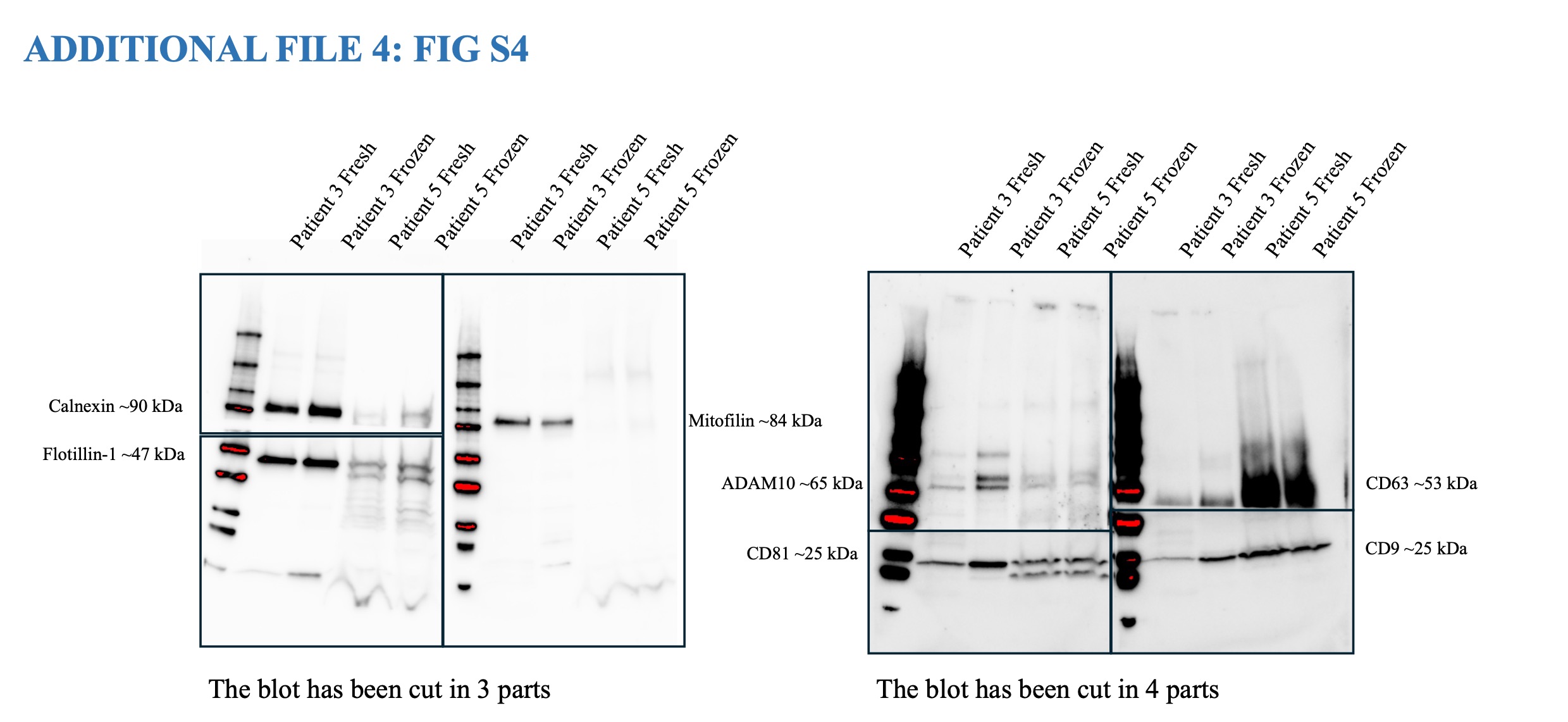

Supplement: Supplementary file 4 — Supplementary Material 4: ADDITIONAL FILE 4: FIG. S4 Uncropped Western Blots Full-length, uncropped Western Blots images corresponding to all cropped blots presented in Figure 3B. [file 12916_2026_4923_MOESM4_ESM.jpg]

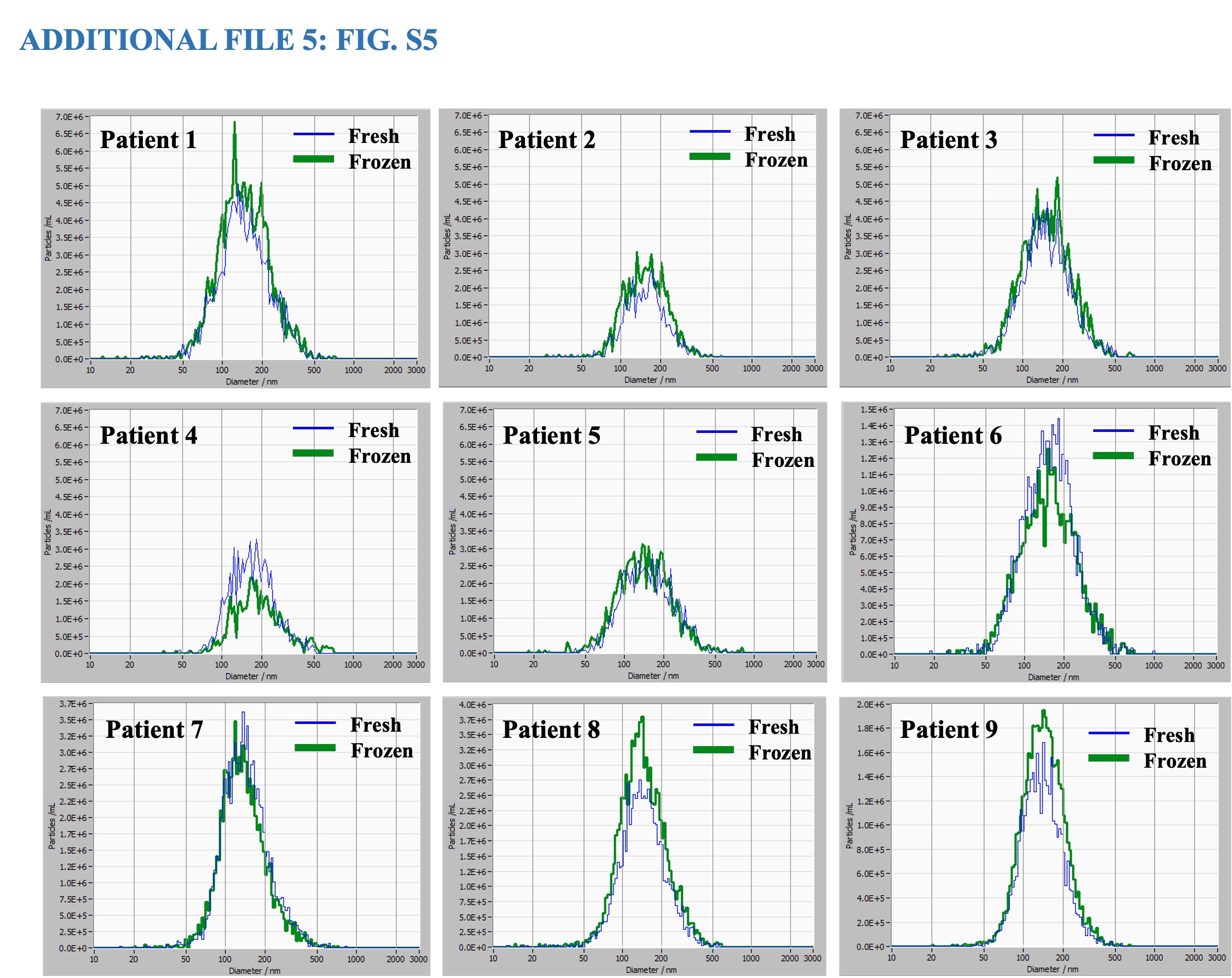

Supplement: Supplementary file 5 — Supplementary Material 5: ADDITIONAL FILE 5: FIG. S5 Size distribution of EVs isolated from each patient obtained using nanoparticle tracking analysis [file 12916_2026_4923_MOESM5_ESM.jpg]

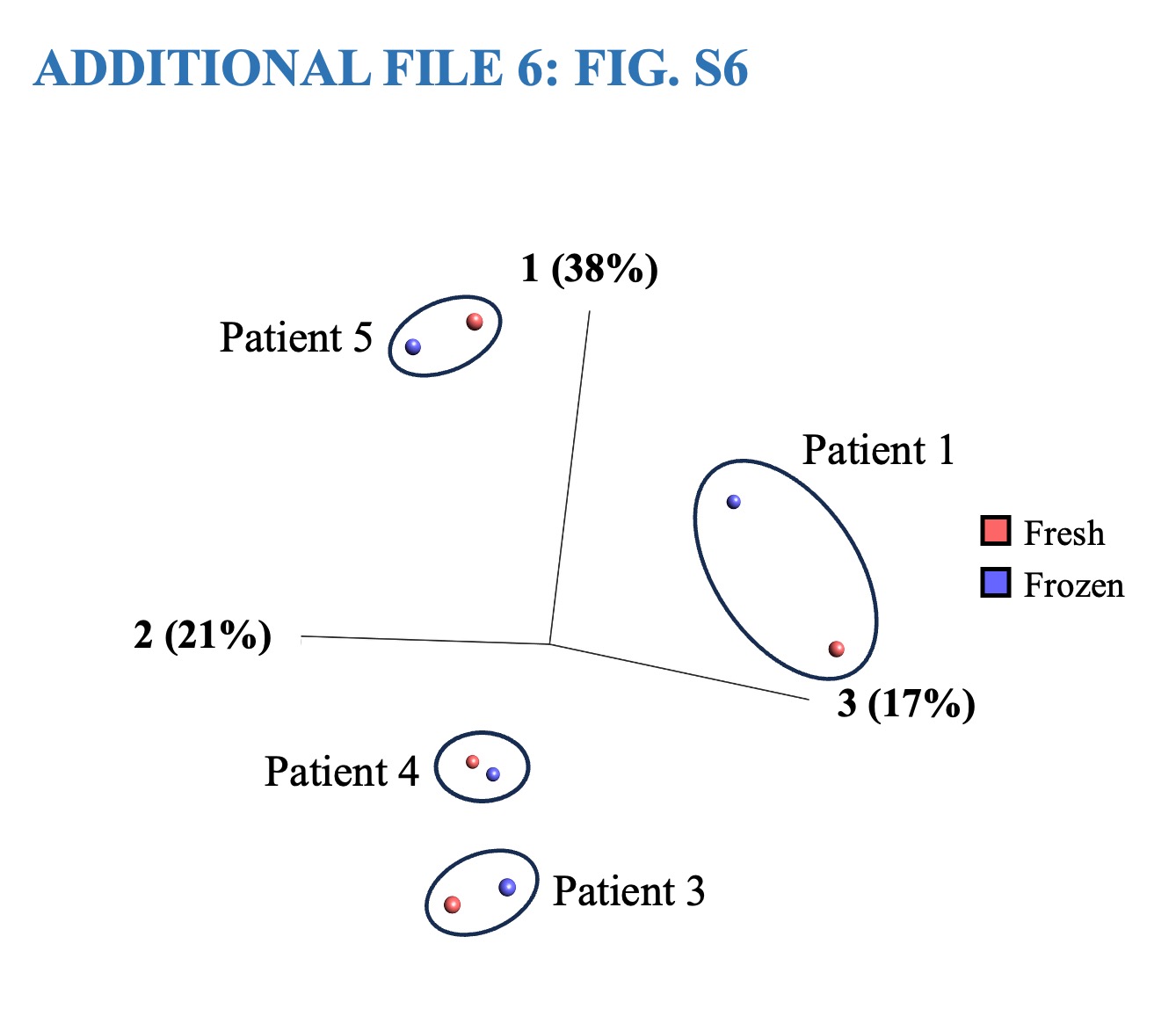

Supplement: Supplementary file 6 — Supplementary Material 6: ADDITIONAL FILE 6: FIG S6 PCA illustrating the relation between fresh (red) and frozen (blue) tissue-derived EVs [file 12916_2026_4923_MOESM6_ESM.jpg]

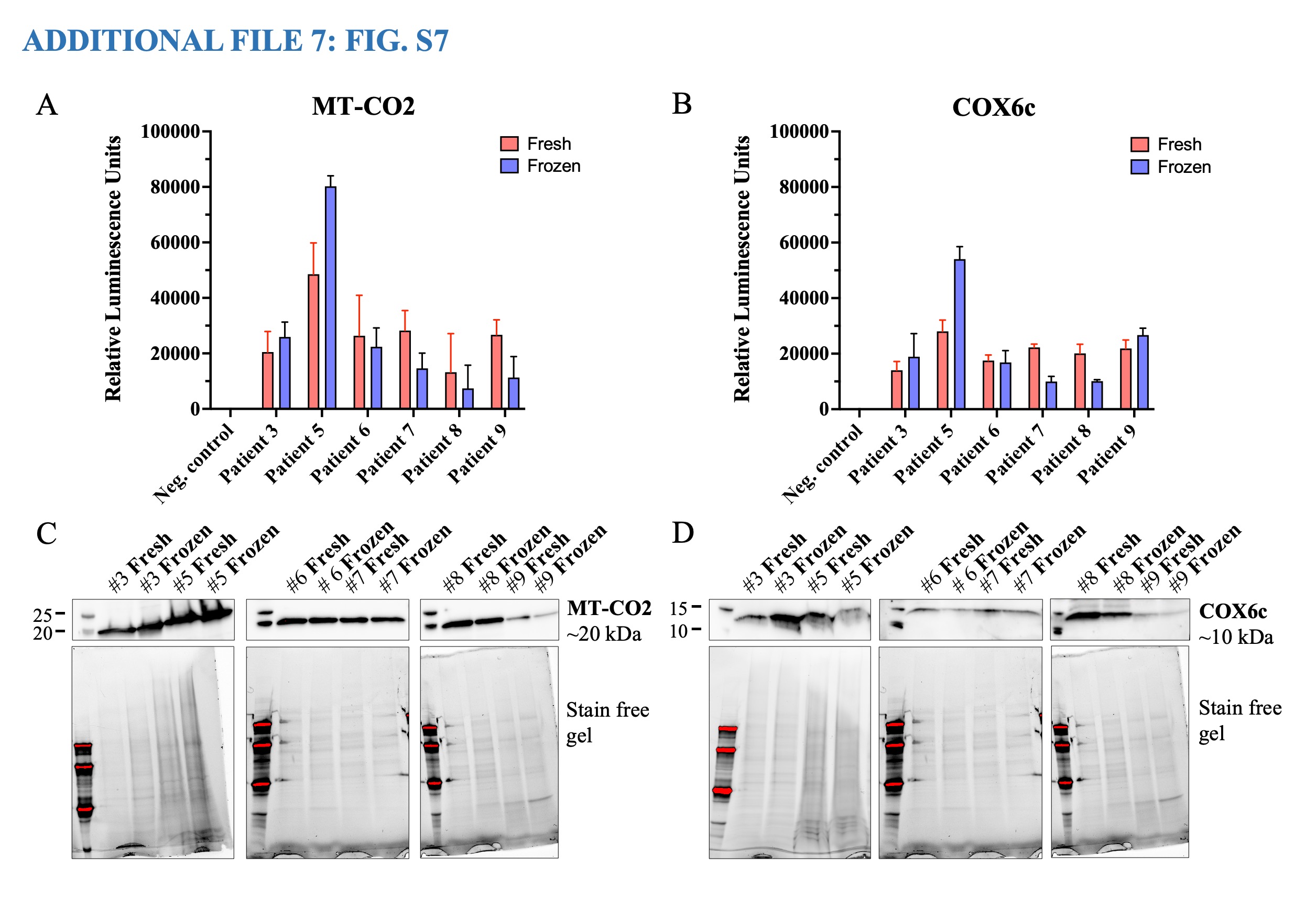

Supplement: Supplementary file 7 — Supplementary Material 7:ADDITIONAL FILE 7: FIG. S7 MT-CO2 and COX6c expression in fresh and frozen EVs [file 12916_2026_4923_MOESM7_ESM.jpg]

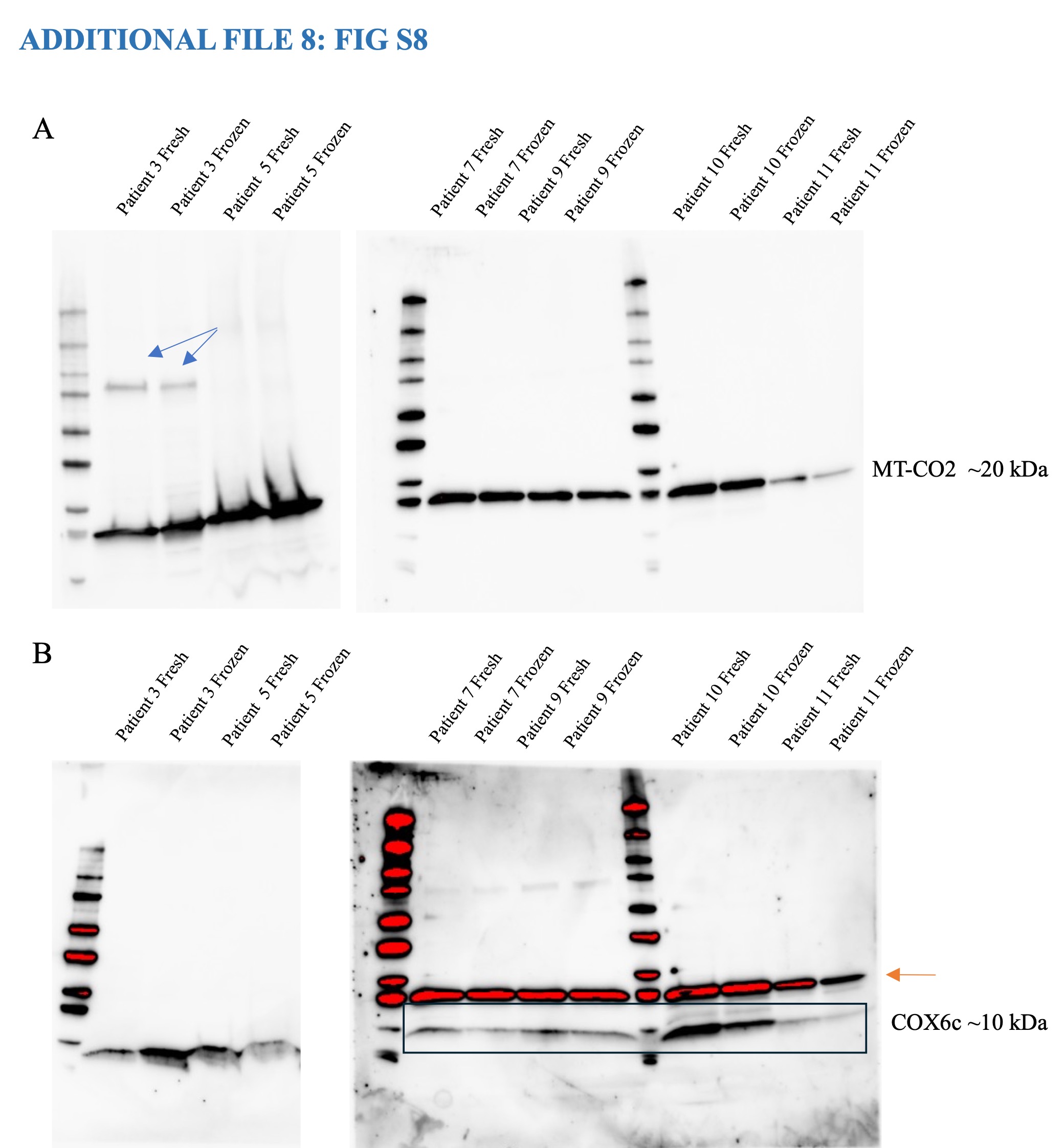

Supplement: Supplementary file 8 — Supplementary Material 8: ADDITIONAL FILE 8: FIG. S8 Uncropped Western Blots. Full-length, uncropped Western Blots images corresponding to allcropped blots presented in ADDITIONAL FILE 7: Fig. S7C and D [file 12916_2026_4923_MOESM8_ESM.jpg]

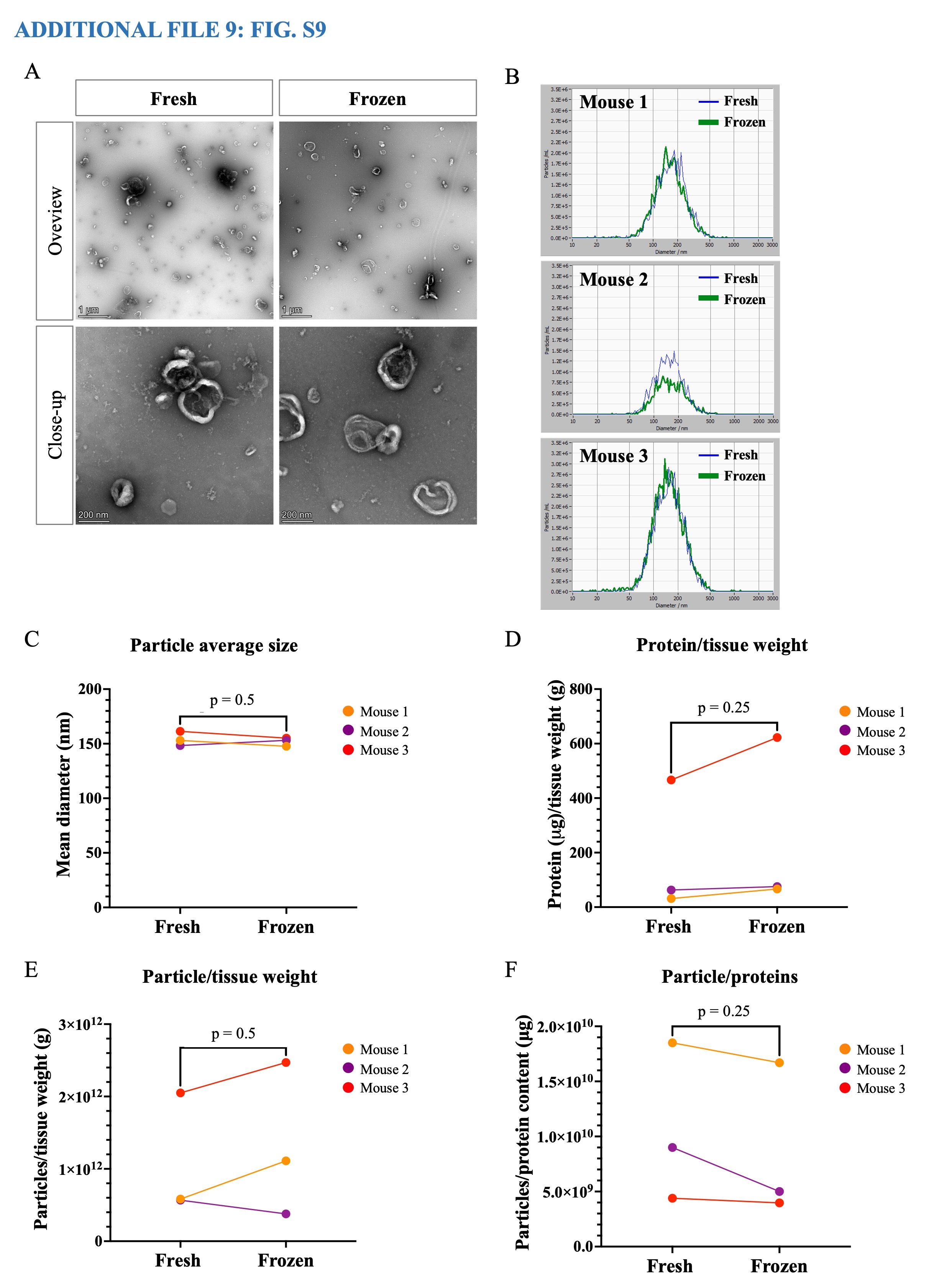

Supplement: Supplementary file 9 — Supplementary Material 9: ADDITIONAL FILE 9: FIG. S9 Characterization of the EVs isolated from the fresh and frozen mouse melanoma tissues. [file 12916_2026_4923_MOESM9_ESM.jpg]
