# Supplementary material for: Frozen melanoma tissues yield extracellular vesicles with preserved diagnostic and immunogenic properties
Source: BMC Med. 2026 May 21;24:325. doi: 10.1186/s12916-026-04923-8 (PMC13198046; doi:10.1186/s12916-026-04923-8)
Supplement: Supplementary file 10 — Supplementary Material 10: ADDITIONAL FILE 10: TABLE S1 Description of the 11-plex Tandem Mass Tag (TMT) labeling [file 12916_2026_4923_MOESM10_ESM.docx]

**Additional file 10 - Table S1:** Description of the 11-plex Tandem Mass Tag (TMT) labeling

EVs were isolated from human melanoma metastatic tumor tissues resected from four different patients.

Immediately after the tumor resection, the melanoma tissues were weighed and divided into two equal portions. One portion was immediately processed (fresh samples), while the other portion was frozen on dry ice (frozen samples). The latter was kept for 2 weeks at –80°C and then processed for EV isolation. Each sample was run in the same TMT set. The layout of the set is shown.

|  | **1** | **2** | **3** | **4** | **5** | **6** | **7** | **8** |
| --- | --- | --- | --- | --- | --- | --- | --- | --- |
| **sample** | Patient 1 Fresh | Patient 1 Frozen | Patient 3 Fresh | Patient 3 Frozen | Patient 4 Fresh | Patient 4 Frozen | Patient 5 Fresh | Patient 5 Frozen |
| **label** | 127N | 127C | 128N | 128C | 129N | 129C | 130N | 130C |
