# Supplementary material for: Frozen melanoma tissues yield extracellular vesicles with preserved diagnostic and immunogenic properties
Source: BMC Med. 2026 May 21;24:325. doi: 10.1186/s12916-026-04923-8 (PMC13198046; doi:10.1186/s12916-026-04923-8)
Supplement: Supplementary file 14 — Supplementary Material 14: ADDITIONAL FILE 14: TABLE S5 List of the 3 mitochondrial proteins enriched in EVs from frozen versus fresh tissues [file 12916_2026_4923_MOESM14_ESM.docx]

**Additional file 14 - Table S5:** List of the 3 mitochondrial proteins enriched in EVs from frozen versus fresh tissues.

The list shows the mitochondrial outer membrane proteins that were enriched in EVs from frozen tissues with a fold change > 1.5 but not significantly different (p-value > 0.05).

| **Accession ID** | **Description** | **Gene name** | **Log_2_ (Fold change)** | **P-value** |
| --- | --- | --- | --- | --- |
| O94826 | Mitochondrial import receptor subunit TOM70 | TOMM70 | -0.701 | 0.353 |
| Q8TB36 | Ganglioside-induced differentiation-associated protein 1 | GDAP1 | -0.734 | 0.53 |
| Q96GF1 | E3 ubiquitin-protein ligase RNF185 | RNF185 | -0.660 | 0.156 |
